# A general and Robust Ray-Casting-Based Algorithm for Triangulating Surfaces at the Nanoscale

**DOI:** 10.1371/journal.pone.0059744

**Published:** 2013-04-05

**Authors:** Sergio Decherchi, Walter Rocchia

**Affiliations:** Department of Drug Discovery and Development, Istituto Italiano di Tecnologia, Genoa, Italy; King’s College London, United Kingdom

## Abstract

We present a general, robust, and efficient ray-casting-based approach to triangulating complex manifold surfaces arising in the nano-bioscience field. This feature is inserted in a more extended framework that: i) builds the molecular surface of nanometric systems according to several existing definitions, ii) can import external meshes, iii) performs accurate surface area estimation, iv) performs volume estimation, cavity detection, and conditional volume filling, and v) can color the points of a grid according to their locations with respect to the given surface. We implemented our methods in the publicly available NanoShaper software suite (www.electrostaticszone.eu). Robustness is achieved using the CGAL library and an *ad hoc* ray-casting technique. Our approach can deal with any manifold surface (including nonmolecular ones). Those explicitly treated here are the Connolly-Richards (SES), the Skin, and the Gaussian surfaces. Test results indicate that it is robust to rotation, scale, and atom displacement. This last aspect is evidenced by cavity detection of the highly symmetric structure of fullerene, which fails when attempted by MSMS and has problems in EDTSurf. In terms of timings, NanoShaper builds the Skin surface three times faster than the single threaded version in Lindow et al. on a 100,000 atoms protein and triangulates it at least ten times more rapidly than the Kruithof algorithm. NanoShaper was integrated with the DelPhi Poisson-Boltzmann equation solver. Its SES grid coloring outperformed the DelPhi counterpart. To test the viability of our method on large systems, we chose one of the biggest molecular structures in the Protein Data Bank, namely the 1VSZ entry, which corresponds to the human adenovirus (180,000 atoms after Hydrogen addition). We were able to triangulate the corresponding SES and Skin surfaces (6.2 and 7.0 million triangles, respectively, at a scale of 2 grids per Å) on a middle-range workstation.

## Introduction

Many scientific disciplines that study systems at the nano- and meso- scales, e.g. biophysics, nanotechnology, and material sciences, often represent matter as a continuum rather than in its full atomistic detail. These continuum models make frequent use of surfaces to separate regions that can be described as homogeneous with respect to some property of interest. An example of this can be found in computational biophysics, where an electrostatic continuum description of charged molecular systems in aqueous solution is often performed to estimate the reaction of both the solute and the solvent to the local electric field. This can lead to deep insights into the nature of biological phenomena such as molecular recognition [1]. In this and in many other contexts, it is highly desirable to have a physically grounded surface definition that also permits a fast and accurate implementation.

If we focus on this type of application, we observe that several models have been used to define a proper molecular surface that separates high (solvent) from low (solute) dielectric regions. This information can be then fed, for instance, to a Poisson-Boltzmann solver, consistently with the Debye-Hueckel theory of weakly interacting electrostatic systems. Among these models, the simplest are the Van der Waals Surface (VdWS) and the Solvent Accessible Surface (SAS). The most commonly adopted is the Connolly-Richards [2] Solvent Excluded Surface (SES), which was first implemented by Connolly [3] in 1983. Finally, the minimal molecular surface results from the minimization of a specific functional [4]. Two other definitions come from the computer graphics field and have been created chiefly for visualization purposes. These are the Gaussian surface [5] (also known in the Graphics community as the Blobby surface) and the Skin surface [6]–[10]. Some of their strengths and weaknesses have already been reported in [11] and are briefly summarized here.

The VdWS is defined as the union of the spherical atoms that represent a molecule. One value of this surface is that the corresponding area can be analytically calculated as well as its gradient with respect to atom coordinates. One limit that it shares with other definitions is the creation of small interstices inaccessible to water that can, if not cured, be erroneously assigned to the high dielectric region. The SES fills those small voids and surface invaginations of the VdWS, which are so small that the entrance of a spherical water probe is obstructed. This definition is particularly physically sound and also widely used because it connects the concept of accessibility to the size of a water molecule. Suitable algorithms for calculating the SES area and its derivatives are also available [12]. As discussed elsewhere, this definition does not lead to the creation of unphysical voids [11]. In contrast to both the implicit Gaussian surface [5] and the Skin surface [6], one of the issues with the SES is that its area is noncontinuous with respect to atom positions [11], [13]. As with the VdWS, however, the former two create unphysical voids and tunnels. The Gaussian surface has the further disadvantage that changing its intrinsic parameter, the blobbyness, to modulate its concave regions and voids has unwanted repercussions on the convex parts [11].

Several existing algorithms adopt different *ad hoc* solutions to build the above-mentioned surfaces. They often achieve good performance at the price of low flexibility. The MSMS package [14] is one of the most used and most efficient. In this package, the SES is computed in two steps. First, the *Reduced Surface* (which is a sort of skeleton) is built. Then, after every patch of the surface is identified, the triangulation is performed. In the first phase, only the skeleton is computed. Predefined templates are then used to obtain the final triangulation. This approach is thus particularly fast. Other approaches expressly designed for the SES (LSMS [15], DelPhi [16], MEAD [17]) perform the computation using a 3D grid. Recently, an elegant approach was proposed by Xu et al. [18], where specialized operators based on the *distance transform* enable the triangulation of VdWS, SAS, and SES via the Marching Cubes algorithm [19]; a similar approach is discussed by Kim et al. [20].

Two conclusions can be drawn from these premises. The first is that it is difficult and possibly ill-advised to search for the *absolute* molecular surface definition. This is because different models can be superior in different contexts. Second, being tailored to a specific surface definition limits the flexibility and hinders the creation of a general standardized framework that could help explore and compare novel models. For these reasons, it is desirable to devise an approach potentially able to cope with existing as well as new possible molecular surface definitions and, more generally, with arbitrarily defined complex surfaces, while at the same time providing an implementation which is computationally efficient for practical applications.

In this paper, we propose a framework for processing an arbitrarily defined surface under the only hypothesis that it is closed and under the fairly reasonable assumption that a surface/ray intersection algorithm is available or can be designed. All of the previously mentioned surface definitions, for instance, meet these requirements and can be processed by this technique. It is hard to think of a physical model that does not fulfill them. By *closed*, we mean that the surface is manifold. This property is met by all the surfaces enclosing a given space region. It includes peculiar geometries such as the surface of a torus, which can be correctly processed with our approach. It excludes, for example, a triangulated surface that has some missing triangles.

At present, our framework includes: i) a *build-up* part, where the shape of the surface is calculated, analytically if possible, ii) a *ray-casting* part, where grid-consistent rays are cast, corresponding intersections with the surface are collected, and enclosed volume is estimated, iii) a cavity detection part, where identified cavities are possibly removed according to their volume or shape, iv) a *Marching Cubes* part, where the surface is triangulated consistently with previous cavity detection/removal and the corresponding surface area is calculated, and v) a projection part, where a subset of the grid points are projected onto the surface. During step ii), a specific ray casting can be performed to color an underlying grid for any kind of purpose, such as PDE solution. For sake of clarity, throughout the paper we will refer to steps i), ii) and iv) as *surfacing* and to steps ii) and iv) as *triangulation*.

In order to prove the effectiveness of our approach, we implemented our algorithms in a C++ portable software suite of tools, which we called NanoShaper, and tested it on a set of relevant biological applications. Our implementation of the framework is endowed with all of the above-mentioned functionalities. It builds the most widely adopted molecular surface definitions for biomolecular systems, either analytically, for the VdWS, the SAS, the SES, and the Skin surface, or numerically for the Gaussian surface. It can import a digitalized closed surface in mesh format (e.g. MSMS.vert and.face files, Geomview.off, and.ply format) in case the user wants to make a grid-consistent re-triangulation or other processing tasks. Triangulated mesh is exported in the GeomView format. In NanoShaper, all computations are done in double precision and, in the build-up part, the full patch trimming information is always derived. The ray-casting part has been parallelized on cpu-based shared memory architectures via multithreading. NanoShaper output can be used for visualization and further computation purposes, including, but not limited to, Finite Difference schemes, Finite Element Methods (after further tetrahedrization), and Boundary Element Methods. In order to test this functionality, we also realized a pluggable version and integrated it with the DelPhi [16], [21] Poisson-Boltzmann equation solver so as to calculate the electrostatic component of the solvation free energy of macromolecules in solution.

## Methods

### Inspecting a Manifold Surface via Ray Casting

**Figure 1:**
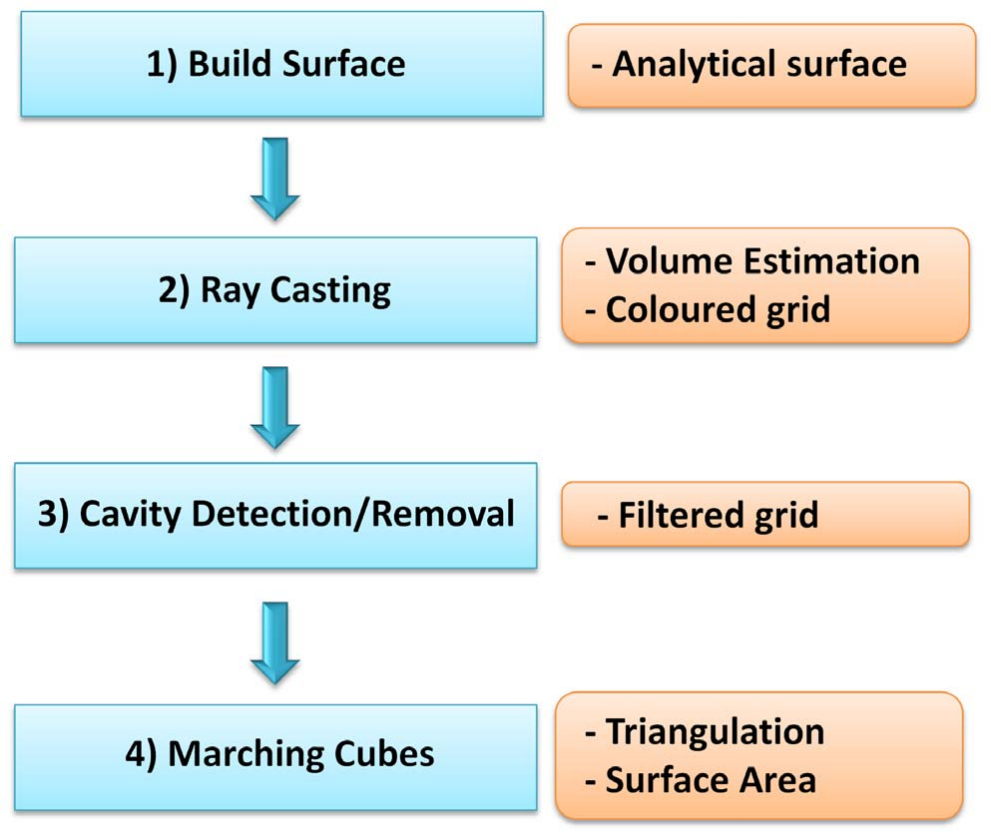
Surfacing Steps. On the left are the steps involved in the surfacing pipeline. On the right are the corresponding outcomes. 1) The surface is computed or externally loaded, 2) the surface is ray-cast, 3) cavities are detected and possibly removed, 4) the surface is triangulated.

Ray casting is a well-known and powerful tool in the graphics community. One advantage of ray casting is that the surface/ray intersection can often be computed analytically without any discretization of the scene. Recent works show how inherently parallel and possibly stochastic approaches can be used to estimate the molecular surface area [22], [23]. In particular, the recent work of Phillips et al. showed how ray casting can be used to analyze the internal region of a closed surface [23]. The basic idea consists in subdividing the space in a 3D cubic grid and then shooting rays either regularly or randomly from one side in order to explore the system. Elaborating on this idea, we cast a regular set of rays and calculate their intersections with the surface. To avoid confusion, we point out that in this work we employ *volumetric ray casting*. In this procedure, all the intersections of a ray and the target are calculated, not just those that are visible by the observer. Our rays have a further peculiarity: each of them follows a coordinate direction so as to provide grid-consistent information. The intersections are calculated analytically when possible, i.e. when an analytical expression is available of the various patches that constitute the surface. Assuming that the grid encloses the entire system, these rays both start and end outside the surface, and forcedly make an even number of intersections with the surface itself. We exploit this fact to generate a sort of checksum, to verify whether an intersection has been missed due to numerical inaccuracy. The intersections identify points that lay virtually exactly on the surface and that can be used for triangulation and successive area estimation. Moreover, the obtained information is used to get volume estimation by a proper discretization of the corresponding triple integral and, in cavity detection, by means of a *floodfill* algorithm [23]. As will be detailed later, in [23], the surface area estimation is sensitive to the particular direction of the rays. The presented framework overcomes this limitation. This is because our rays are cast in three orthogonal directions so as to capture the whole surface topology and details. We organize the rays’ direction and origin to obtain a coloring of the grid that allows, for instance, the solution of a Partial Differential Equation (PDE) within a finite difference scheme without any further processing. Precision and numerical robustness of the method are guaranteed by the introduction of an analytical version of the Marching Cubes (MC) algorithm [19], and by the use of the Computational Geometry Algorithms open-source Library (CGAL) to compute geometrical primitives such as the Weighted Delaunay Tetrahedralization, which allows the treatment of singular geometric configurations where other implementations, which rely on floating point numbers only, may fail. As a final note, the algorithm is inherently and easily parallelizable because rays are independent of one another. The overall flowchart is given in Figure 1. In the following section, we describe how we build the molecular surfaces and then discuss the ray-casting algorithm.

### Molecular Surface Construction

Here, we describe the algorithms that we devised to build the most widely used molecular surface definitions, and that have been implemented in NanoShaper. More details and corresponding figures are reported in the Supporting Information (SI).

#### The Connolly-Richards solvent excluded surface, VdWS and SAS

The SES consists of several components. The main component can be imagined as the surface delimited by a spherical probe rolling over the solute. The others correspond to the internal cavities where the same rolling process occurs. Convex regions of the SES generate spherical patches while concave regions lead to patches that are portions of either spherical or toroidal surfaces. We use alpha shapes theory [24] to build a topologically correct SES. Details on how to compute patch equations, trimming spheres, and trimming planes can be found in [25], [26]. We used CGAL to compute the Weighted Delaunay Tetrahedralization and the alpha shape. To achieve both a fast and reliable computation, we used the *exact predicates and inexact construction kernels* of CGAL during both the Weighted Delaunay Tetrahedralization and the alpha shape computation. This setting uses adaptively floating and fixed point arithmetic. It allows a good tradeoff between accuracy and speed. To treat degenerate configurations, we randomly perturb atom positions on each coordinate with a maximum value of 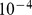
Å. This value was chosen because it is the largest nonsignificant digit in the PDB format [27]. While the ray-sphere intersection can easily be computed, the ray-torus intersection is worked out by explicitly solving the corresponding polynomial root-finding problem. For this, we used a Sturm sequences root solver [28].

With the same algorithm, the VdWS and the SAS can also be computed. This is because the first corresponds to the SES with null probe radius, and the second is the VdWS of the system when all of the radii have been increased by the probe radius value.

#### The gaussian surface

Defined as the isosurface of a scalar field, it can be expressed as the sum of atom-centered gaussians:
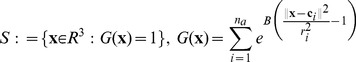
(1)where 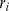 is the radius of the i-th atom, 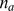 is the number of atoms, 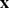 is the current point on the surface, 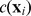 is the 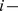
th atom center, and 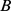 is a parameter (the *blobbyness*) that controls the surface roughness as the probe radius does in the SES surface [3].

The *Gaussian* surface is easy to implement because the main computation is the evaluation of a Gaussian kernel function. The surface is differentiable, and free of self-intersections and singularities. Moreover, a Gaussian atomic density representation has some grounds from the physical point of view since it recalls the spherical atomic orbitals. The behavior of this surface has been discussed in [11], and high performance GPU implementations were developed in [29] and [30].

To manage this surface, the following steps are performed: scalar field computation, Marching Cubes triangulation, and ray casting. In principle, the last step is not necessary. This is because, given the scalar field, a coloring of the grid is already available. We perform this last step only if we need to modify the triangulation of the mesh to make it grid-conforming or to compute the volume enclosed by the surface. In the current implementation, in order to speed up the calculations, the scalar field is computed in an atom-centered fashion, employing a cut-off of 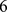
Å.

#### The skin surface

The *Skin* surface was formally defined in [6]. The salient features of this surface are:

- It can be decomposed in a finite set of trimmed quadric surfaces.
- There exist fast combinatorial algorithms (i.e. the Weighted Delaunay Tetrahedralization) to build it.
- Pathological configurations leading to discontinuity of the normal vector are extremely limited.
- Its area is continuous with respect to atom positions and radii.

The *Skin* surface can be built starting from a set of weighted points 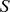:
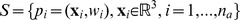
(2)and a shrink factor 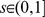. When representing a molecule, the points 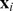 are the atom centers, 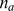 is the number of atoms, and 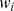 are the weights. The weights 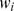 are defined as:
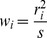
(3)where 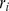 is the i-th atomic radius. More details as well as a graphic example are given in the SI.

Although they share some of their mathematical foundations, computing the *Skin* surface is more expensive than computing the SES. This fact has already been observed in [31] and it is confirmed and heuristically explained here. Indeed, computing the *Skin* surface requires first the computation of a regular Delaunay Tetrahedralization, whose computational cost is 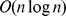 where 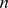 in this case is the number of atoms. After this step, the computation of the so-called *mixed complex* (see SI) and the patches are required. Finally, either a volumetric or a mesh representation must be created.

### Ray Casting for Triangulation and Analysis

As can be seen in Figure 1, the first step consists in building the description of the surface (e.g. the set of patches) according to, for instance, one of the previously discussed algorithms. This can be done analytically, by computing a set of equations and trimming solids, for the Skin surface and the SES, VdWS, and SAS. It has to be done numerically if, as with the Gaussian surface, the surface is defined as the isocontour of a scalar field.

**Figure 2:**
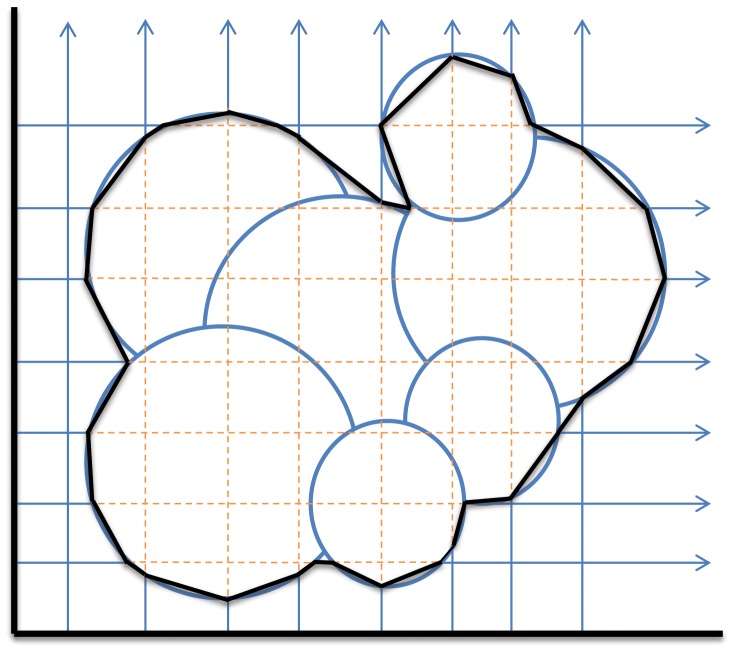
Ray-casting procedure. Rays are cast from each coordinate plane. In this 2D sketch, a coordinate plane is represented by a black line. For rays, orange segments correspond to the internal region. In black is the triangulation deduced from ray-surface intersections.

Once the surface patches are obtained, the ray casting is performed. A box with sides parallel to the coordinate plane is created around the system, and a first group of rays, which we call Grid Rays, are cast from each point of the 2D grids created on three orthogonal faces of the box (see Figure 2). Shooting from the three directions provides the in/out information not only at the grid cubes’ center, but also at the center of their faces, as is often required by discretized PDEs. In addition to provisioning the construction of the volumetric map to be used directly for equation solution, this phase is instrumental for cavity detection/removal and for volume estimation. Evidently, surfaces that are already triangulated can also be ray-traced with the strategy just described. In order to intersect rays and triangles, we used the routine described in [32], which proved to be fast and reliable.

While computing surface/ray intersections, it would save a lot of computations to know *a priori* which patches that the ray will encounter. For this reason, together with the baseline 3D grid, three auxiliary and possibly less dense 2D grids, one for each coordinate plane, are used to collect the information needed to rapidly retrieve the patches during ray traversal of the grid. This acceleration data structure is built before doing ray casting. It employs a bounding box for each surface patch to generate lists of pointers at a superset of the patches that will be encountered during ray traversal. More details are given in SI.

#### Volume estimation

We perform volume estimation according to the procedure detailed in [23]. We take advantage of the fact that our ray casting is done in the three orthogonal directions to see whether averaging the values obtained over the three corresponding discretizations of the following integral will improve the accuracy of volume estimation:
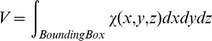
(4)where 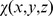 is a suitable indicator function whose value is 1 inside the volume and 0 elsewhere. Results show that the one-directional procedure already provides a good accuracy level, and that three-direction averaging is not needed.

#### Grid consistent triangulation and area estimation

**Figure 3:**
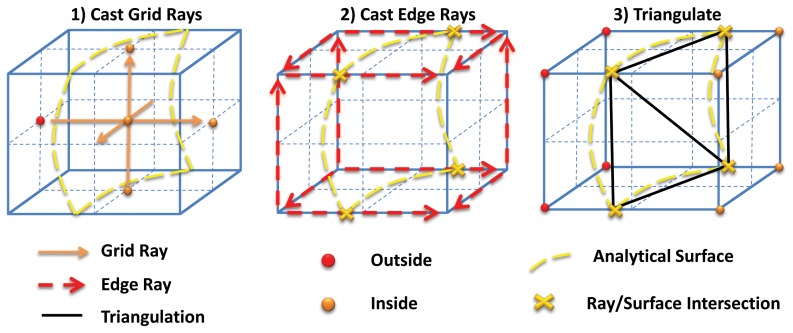
Ray casting on the surface. In (1), rays are cast along the centers of the grid cubes to get the inside/outside information. Then (2), Edge Rays are cast and analytical intersections with the surface are calculated, inferring the inside/outside status of the vertices of each cube. Finally (3), the triangulation is performed employing the Marching Cubes triangulation maps.

A second group of rays, which we call Edge Rays, are also cast along the grid cube edges. These rays are cast in order to categorize the vertices of the grid cubes and to collect the ray/surface intersections along grid edges (see Figure 3).

The final outcome of this phase is a grid that is populated with all the surface information needed by the Marching Cubes triangulation tables [19], with the additional advantage that all the computed intersections can be analytical. In fact, in the original Marching Cubes algorithm, one computes a scalar function on a grid and then performs an iso-extraction process by employing a set of pre-defined triangulation tables. Marching Cubes deduces the positions of the vertices of the triangulation by an interpolation method, usually the linear one. In other words, in order to apply the Marching Cubes triangulation rules, one needs a scalar map and an iso-value to estimate the position of each vertex by interpolation. In our scheme, ray casting is used to generate the in/out information that can be used as a scalar map. The vertex positions are not deduced by interpolation along the edges, but are given by the (possibly analytical) intersections of the surface with the rays. For this reason, our method could be termed *‘Analytical Intersections Marching Cubes’*. This is because each vertex of the triangulation is obtained not by interpolation but by directly sampling the surface. It thus belongs to the surface up to numerical inaccuracy. Interestingly, both Marching Cubes and ray casting are rendering methods used to triangulate and visualize surfaces. Within our protocol, they are not treated as alternatives. Instead, they cooperate to produce an accurate, simple, and parallel triangulation algorithm. In the end, we retain the accuracy of ray casting to get the intersections, and we retain Marching Cubes’ ability to triangulate the surface in order to get a usable mesh of the model. The reader could argue that shooting the Edge Rays is not needed to get a triangulation, because Grid Rays can already collect all the needed information. This is correct up to the observation that, in order to get triangles that are not cut by the grid cubes, one has to re-triangulate internally to each grid cube. Thanks to Edge Rays, we do not need this second step. In several numerical applications, such as PDEs numerical solution, it is important to have a simple method to estimate the area of the surface restricted to a grid cube. This *grid-consistent* triangulation method, directly and without any post-processing, makes this possible. The surface area of each grid cube can be estimated by easily summing up the areas of each triangle in each grid cube.

If one wants to trade accuracy for speed, we can envision a faster and more memory-efficient variant of this method. We will refer to it as *‘Bisecting Marching Cubes’*. Here, only Grid Rays are cast. The information concerning cube vertices is inferred by the neighboring grid cube centers. In this way, the virtually exact information on the vertices is lost but the ray-casting cost is reduced to one third. Memory requirements are also significantly reduced. This is because vertex arrays are no longer allocated. Vertices are rather created on the fly as is usual in the Marching Cubes algorithm.

At the end of any of the described triangulation routines, a laplacian smoothing (low pass mesh filter) can be applied in order to improve the mesh quality. One of the issues that may occur during ray casting is that, due to numerical inaccuracy in the intersection routine, some patches are missed or some intersections are inaccurate so that the total number of intersections per ray is odd. In visualization, this leads to a possibly minimal imperfection in the frame. However, this cannot be tolerated in numerical applications, as the PDE solution we are considering in this work. To address this issue (occurring, for instance, when triangles are almost degenerate), we adopt a checksum criterion followed by an *ad hoc* procedure. When a ray is cast, the total number of intersections is always checked. When there is evidence of an anomaly (an odd number of intersections), the ray is recast by employing the same little (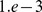) random perturbation to both the starting and end points of the original casting path. If, after 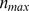 trials, currently 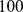, the problem is still present, then the last stable traced path is copied to the current one. In this albeit extremely rare case, the surface is assumed to be locally constant, minimizing the distortion due to the inaccuracy. In practice, as detailed in the results, we observed only a few cases of such instabilities. These were always due to bad triangles or degenerate patches, which were successfully cured with this protocol.

#### Cavity identification and filtering

As anticipated, our triangulation is also consistent with cavity detection and removal. Indeed, we perform cavity detection on the center of the grid cubes by using a floodfill procedure [23]. At the same time, we estimate the volume of each cavity. In many cases, there is the need to eliminate, i.e. to fill, the cavities that are smaller than a given threshold, e.g. if their volume is smaller than that of a water molecule. Here, this is done by simply toggling the status of grid points in that cavity from external to internal. Cavity removal can sometimes lead to inconsistent configurations, more details concerning this occurrence and how we cope with it are given in the SI.

Performing MC after cavity removal leads to a mesh that is consistent with the system after cavity filtration. This prevents doing any successive fixing of the mesh. This functionality can be particularly useful for complex surfaces such as the Skin or for implicitly defined surfaces where spurious and unforeseen interstices can appear [11].

## Results

We first validate our implementation of the described methods by checking the stability and robustness of volume estimation, area estimation, and cavity identification. This is done at different grid resolutions and using different surface definitions. We then adopt several different figures of merit to compare our framework with the most representative alternatives available in the scientific community. Namely, we consider timing, memory consumption, generality and sensitivity to geometric pathological configurations. Finally, we compare our SES building routine, grid coloring and projection on the surface with the internal routines of DelPhi, a popular, Finite-Difference-based, Poisson-Boltzmann solver [16], [21].

All computations were performed on an 8-core AMD Opteron 2350 (year 2008) workstation with 8 GB of RAM.

### Validation of the Approach

The aim of this section is to test the stability and robustness of NanoShaper when changing grid resolution and surface definition. Figures of merit are robustness with respect to numerical instability, cavity identification and treatment as well as volume and area estimation. For these latter tasks, we specifically consider the Fatty Acid Amide Hydrolase (FAAH), a protein that plays a key role in endogenous pain control and presents quite a number of cavities as well as a narrow and bifurcated site where it exerts its function.

#### Ray-casting numerical instability management

To evaluate the numerical effects of coplanarity between rays and surface patches and those due to degenerate patches/triangles, we computed and ray-cast the triangulated meshes produced by MSMS over the first 1000 entries of the PDB repository. We used a scale of 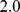 grids per Å and we verified that in 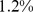 of the cases at least one triangle of the mesh is either coplanar or degenerate. Even in these cases, and using the described random ray re-casting method, our method generated a proper triangulation, so as to always meet the checksum test.

#### Cavity detection

**Figure 4:**
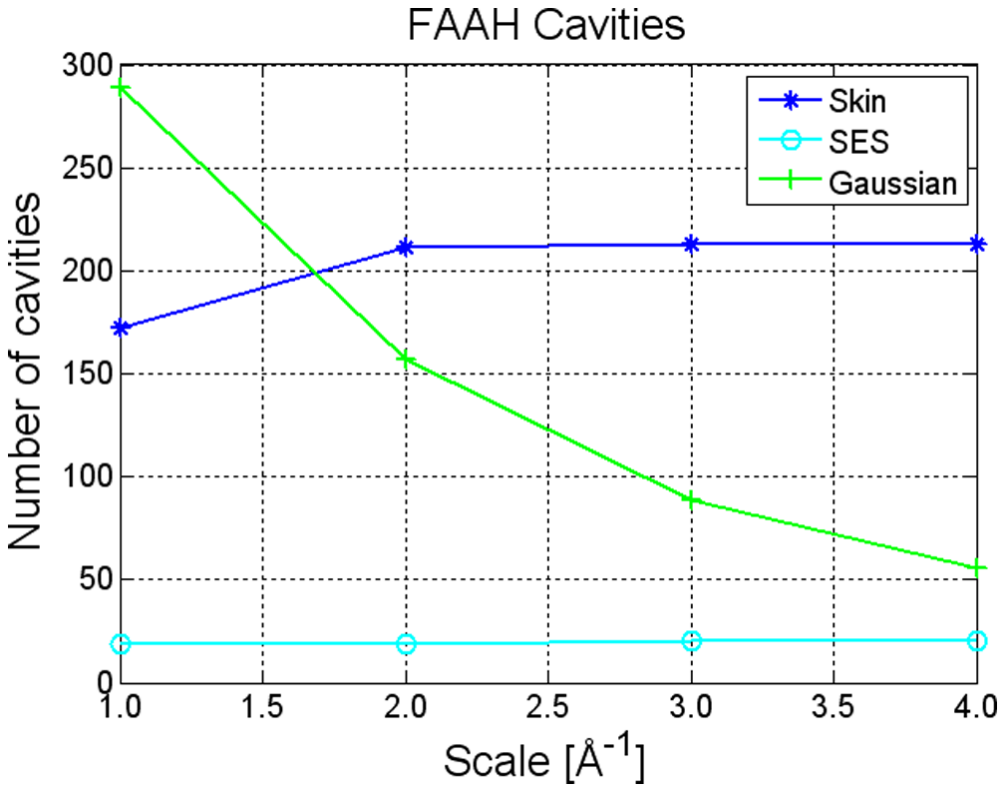
Cavity detection. Cavity detection for NanoShaper-built Gaussian, Skin, and SES.

In this group of experiments, we analyzed the behavior of the cavity detector with respect to grid resolution, using FAAH as a test case. Results indicate that the Skin presents a high number of small cavities that can, however, be reliably detected and are stable with respect to scale changes (see Figure 4). In turn, the Gaussian surface with parameter 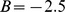 is rich in narrow tunnels. At low resolution, these produce a significant number of false positive voids, which gradually reduce as scale increases. Just as a note, if we change the blobbyness to 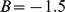, the number of cavities reduces and a more stable behavior is obtained at the price of a deformed surface in its convex regions too. This is consistent with the work of other groups, e.g. [33], that used a value of B able to prevent unwanted voids and tunnels even if the resulting shape was suboptimal, namely too *blobby* to mimic a physically sound solvent/solute interface. There is no need to filter the SES for cavities smaller than a water molecule; this is already granted by the SES definition. Therefore, the actual number of cavities expected for FAAH is around 20. All the others are small interstices that should be eliminated.

**Figure 5:**
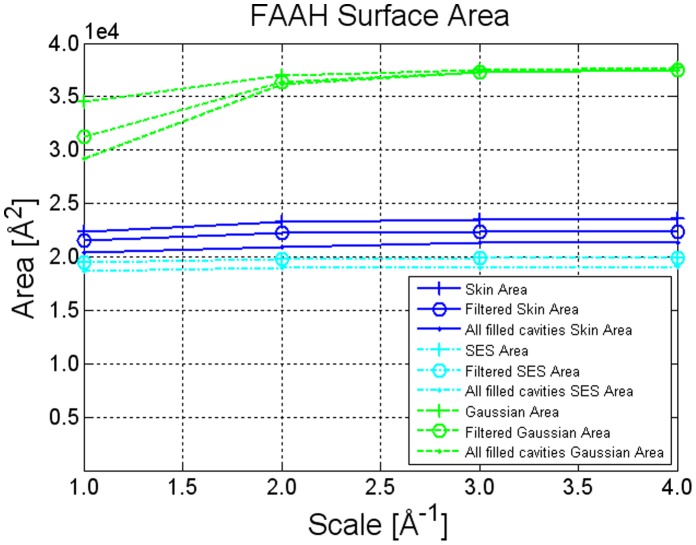
Area estimation: surface definition comparison. FAAH protein surface area estimates in case of Skin (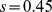), Gaussian 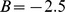, and SES (probe radius 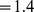 Å), with changing grid resolution and enabling cavity removal.

Cavity detection obviously affects area estimation, since the area of the cavities contributes to the overall surface area. Results in Figure 5 show that, similarly to the case where cavities were absent, a scale of 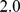 grids per Å is sufficient to achieve a good estimation of the surface area. We also assessed how the surface area is affected by cavity filtering. For every definition, we performed two different cavity removals. First, we removed cavities whose volume was less than 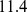 Å^
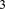
^ as an approximate water molecule volume (in Figure 5; this is called *filtered area*). Second, we removed all the cavities regardless of their volume. As expected, SES area does not change when filtering cavities smaller than a water molecule because such cavities are already filled according to the SES definition.

#### Volume estimation

We first evaluate the sensitivity of the FAAH estimated volume with respect to grid resolution. With Skin surface and SES, the volume was evaluated directly by performing ray casting after computing the surface patches. To test the processing of imported meshed surfaces, we used the mesh produced by the MSMS program (probe radius 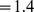
Å and *hdensity = density = 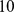
*Å indicating an accurate triangulation) and the Gaussian surface.

**Figure 6:**
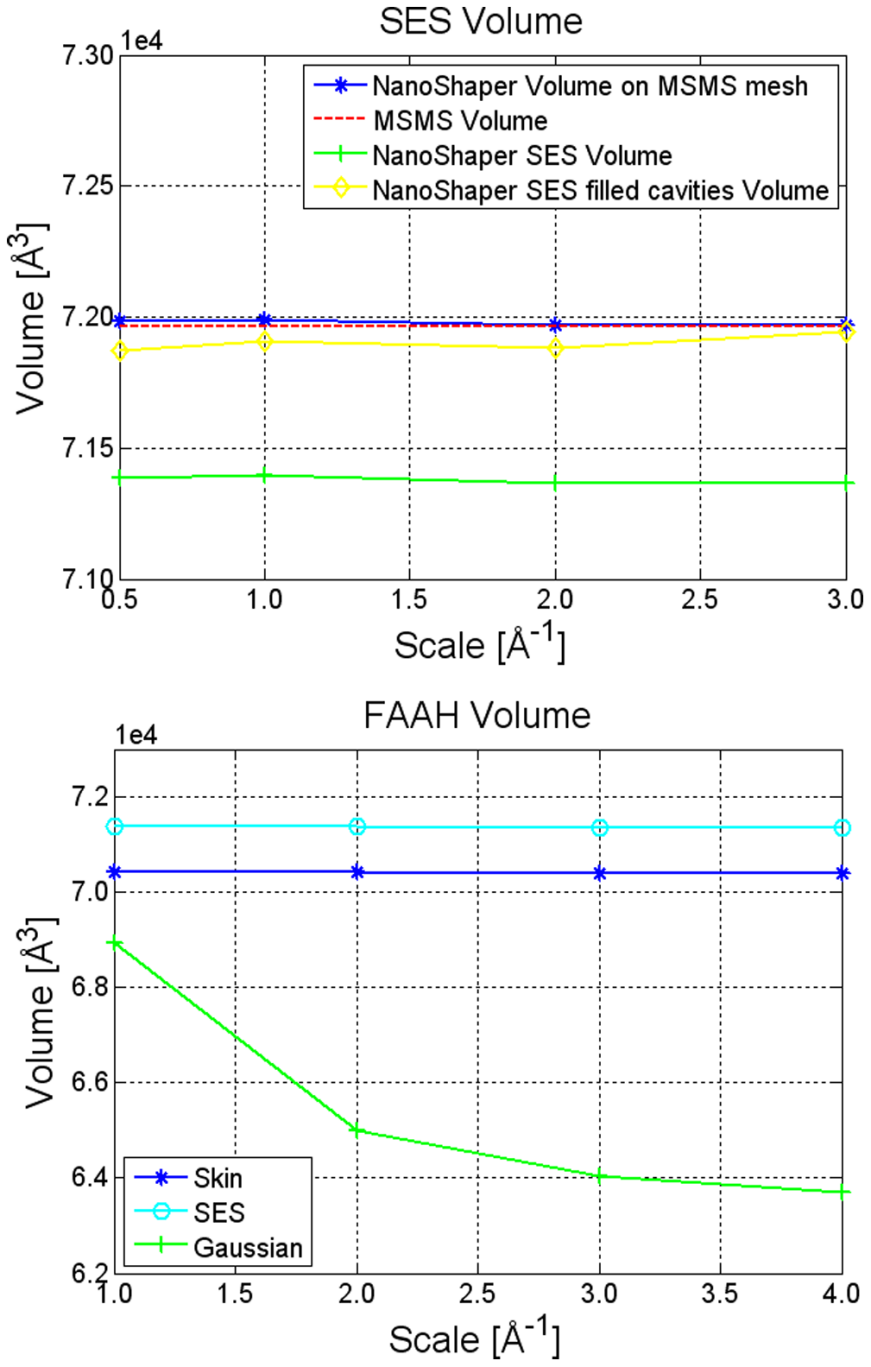
Volume estimation for the FAAH protein. Estimated volume of the FAAH protein at varying grid resolutions and different molecular surface definitions. The upper panel represents a detailed comparison of the SES. Results show that NanoShaper SES after cavity removal is approximately equal to the main component generated by MSMS. In the lower panel for the Skin, we used a value of s = 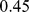, for the Gaussian a value of B = 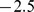, and for the SES a probe radius of 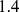 Å.

Figure 6 shows the obtained results. The most stable behavior is exhibited by Skin surface and SES. A scale of 2.0 grids per Å  seems enough to provide a reliable estimation of the volume. These results also indicate that the method is generally stable with respect to grid resolution for all the definitions. Among them, the least stable is the Gaussian surface (for a discussion on the properties of different molecular surfaces see [11]).

Let us now consider the effect of cavities on volume estimation. By default, MSMS calculates only the the main component of the SES and the corresponding volume. In Figure 6, this is called *MSMS volume*. If one considers all the SES components and does not fill any cavity, one gets the actual SES volume. In Figure 6, this is termed *NanoShaper SES Volume* and is the default volume value given by NanoShaper. To have a fair comparison, we also used NanoShaper to calculate the equivalent of the *MSMS volume*. Therefore, we performed cavity detection and summed the volume of each cavity to the *NanoShaper SES Volume*, we termed the result *NanoShaper SES filled cavities volume*. As a further verification, we observed that the volume estimated by ray-casting the MSMS mesh using NanoShaper is in excellent agreement (within 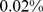) with that calculated by MSMS itself on the same mesh.

### Comparison with Other Surfacing Algorithms

#### Build-up

The aim of this section is to analyze in detail the timing performance of the surface build-up part of the entire surfacing process. Our benchmark consisted of the same set of molecules used in [31] and [25]. In the first columns of Table 1, we compare our results to those obtained in [31] and [25] for the SES. Our comparison both reports the obtained execution time and its adjusted version when taking into account the different hardware used: the ratio between the two architectures has been estimated as 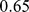 according to the Passmark CPU benchmark [34]. As a result, the SES timings we obtain are similar or only slightly longer. In contrast, the timings for the Skin surface are smaller than in [31], as can be seen in the last two columns of Table 1. In particular, always adjusting for the hardware difference, in the biggest case 3G71, we are roughly 3x faster than the corresponding (single-threaded) run. For these tests we used the same shrinking parameter from [31], namely 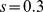; we observed that when changing this parameter to 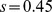 (that we use by default) we obtained almost identical build-up performances, while the number of patches signficantly increased.

**Table 1:** SES and Skin surface: build-up time comparison in seconds (NS stands for NanoShaper and Adj. for adjusted for different architecture performance).

PDBcode	Atoms	SES[31]	SES[25]	SESNS/Adj.	Skin[31]	SkinNS/Adj.
1VIS	2531	0.10	0.08	<1.0	0.45	<1.0
1AF6	10517	0.45	0.36	<1.0	2.11	<1.0
1GKI	20150	0.88	0.77	<1.0	4.18	2.0/1.3
1AON	58870	2.41	2.68	4.0/2.6	9.92	6.0/3.9
3G71	99174	4.49	–	8.0/5.2	18.51	10.0/6.5

Timings for NS have been measured at a resolution that is not accurate below the second, while reported timings for the other approaches have been taken from the respective publications.

These results are interesting because they have been obtained under numerical stability warranties that are not provided in either [31] or [25]. Moreover, we always completely clip our patches and we use double precision in all floating point operations.

#### Ray casting

We measured the speed of the ray-casting routine for meshed surfaces by comparing our approach to that of Phillips et al. [23] on the alcohol dehydrogenase protein (PDB code 1A4U). As in [23], we computed the MSMS mesh and then we ray-cast it to get the volume. With similar settings, a grid of 209 as in [23] (which corresponds to a scale of 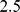 grids per Å), and on our hardware (which is slightly slower than that used in [23]) we obtained an execution time of 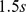, which matches that obtained in [23]. If we additionally triangulate the surface (that is, casting Edge Rays plus performing Marching Cubes), the execution time increases by 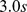, reaching a total of 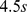.

#### Joint build-up and triangulation

A separate analysis of build-up and triangulation phases turned out to be unviable when considering other software tools as it would have required a reverse-engineering attempt. Therefore, in this section we will compare the construction and triangulation of several molecular surfaces in terms of execution time, required memory, flexibility, robustness, and accuracy in area and volume estimation. We will provide separate figures in terms of different phases for NanoShaper only. In particular, we first compare our Skin surface to that obtained by the CGAL Skin surface module [35] in terms of time and memory performance. Then, our SES surfacing is compared with both EDTsurf [18] and MSMS [14] in terms of accuracy, memory consumption, and execution time. EDTSurf and MSMS are two publicly available tools that not only triangulate but also compute surface area and volume, and detect cavities of the SES. Additionally, MSMS is able to analytically compute SES area.

We put less emphasis on our Gaussian surface implementation since it is not particularly optimized. We refer the reader to the works [29], [30] for high performance Gaussian surface computation implementations.

As a further computational note, we report here the full time needed to compute the surface, consisting in both building the analytical description and performing the ray casting. In the current version of NanoShaper, only ray casting exploits the multi-threading, thus the parallel efficiency is limited by the build-up and marching cubes steps. We only briefly mention that, for the SES case and considering only ray casting, we obtained a very high parallel efficiency. Thanks to hyperthreading, we reached up to a 10x speed-up on our 8-core machine with respect to the single-threaded version.

The experimental setup used to perform the comparisons is as follows:

- We use the same radii used by EDTSurf in their experiments [18]. To do this, we modified EDTSurf so that it exports the radii. We used these latter to build the systems in NanoShaper and MSMS.
- For EDTSurf and NanoShaper, which are grid-based, we set the same scale. For MSMS, to achieve similar resolution, we count the vertex density per unit of area obtained by NanoShaper and we feed MSMS with it.
- MSMS, by default, computes only the outer component of the SES. In contrast, EDTSurf and NanoShaper compute all of them. We thus execute MSMS with the -all_components option to force the computation of all the internal cavities. In the case of the comparison with the CGAL Kruithof algorithm for the Skin, the cavity detection is not performed because this feature is not available in CGAL.
- For the tests, we use a probe radius of 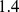
Å for the SES and 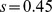 for the Skin surface.
- To perform scalability tests in terms of memory and execution time, we chose one of the biggest PDB structures, namely the fundamental unit of the human adenovirus capsid (PDB code 1VSZ), which, after hydrogen addition, is about 180,000 atoms in size. We subsequently cropped this molecule in subsets of 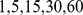,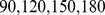 thousand atoms, preserving the original atomic order of the PDB file. To compare the accuracy in the SES construction, we use Calmodulin, FAAH, and the Fullerene buckyball. The first has a cavity-free SES and allows the specific comparison concerning only the main component. The second is a good system for testing how cavities are treated since it has a long and narrow catalytic site. The third is interesting because its highly symmetric geometry constitutes a challenge for the algorithms.
- Execution times are measured as the total time needed to perform a run by the various tools on our benchmark workstation. For the sake of completeness, we note that, unless otherwise stated, the ray-casting routine of NanoShaper was run in multi-threading, while the remaining tasks have not yet been parallelized.

#### Skin: NanoShaper vs the kruithof algorithm

**Figure 7:**
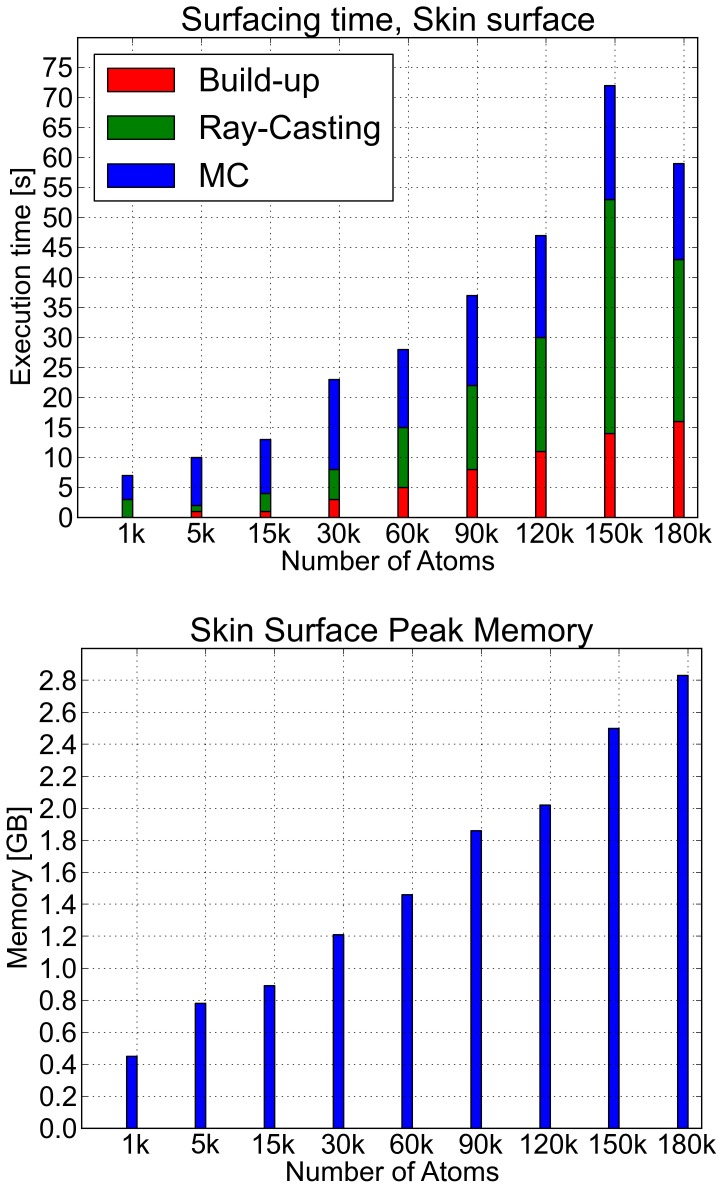
Skin surface: performance. Execution times and memory usage are reported in the upper and lower panels, respectively. The scale for each molecule was set accordingly to that assigned by EDTSurf [18].

The Skin surface Kruithof algorithm in CGAL [9] is an elegant and precise method for building and triangulating the Skin surface, and for guaranteeing the correctness of its topology. In the first graph in Figure 7, NanoShaper timing performance is analyzed with the detailed time required to perform build-up, ray casting, and marching cubes triangulation.

**Figure 8:**
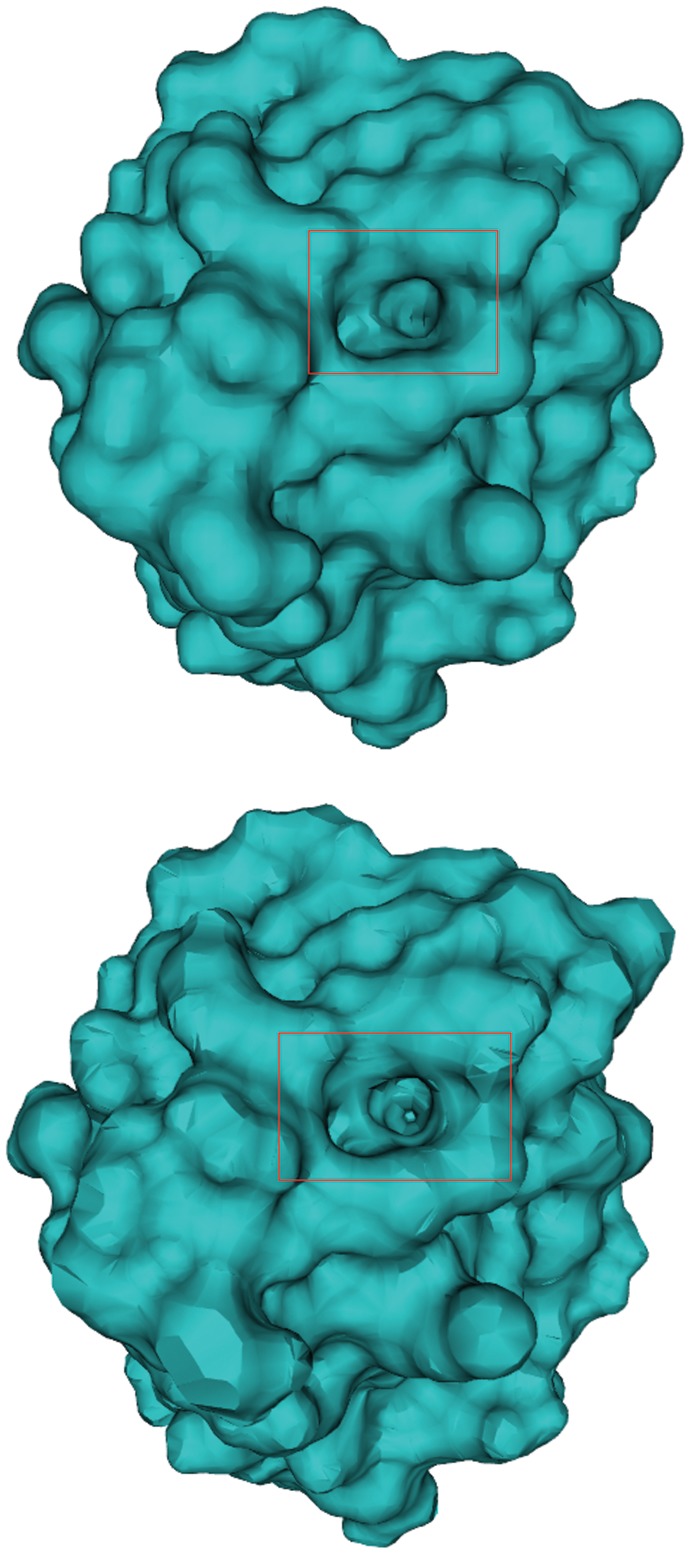
Skin surface: mesh quality. Comparison of the mesh of the Skin surface obtained by our algorithm (up) and CGAL (down) with a small detail highlighted.

Using CGAL Kruithof algorithm on our workstation, we were able to triangulate the Skin surface up to 4000 atoms. For this size, the execution time was 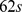, and memory usage was 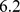
GB of RAM, while NanoShaper took 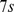 and required 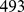
MB. These results can be explained by the way the mixed complex (see SI) is managed: the Kruithof algorithm performs a tetrahedralization of the entire mixed complex to get a topologically correct surface and to recover what he calls “anchor points” (see [9]). In our grid-based approach, we do not analyze all the details of the mixed complex. Rather, it is the user that, by choosing the scale, decides the level of detail needed for the surface. The quality of the triangulation generated by NanoShaper, without any further processing, is however comparable to that of the Skin Kruithof algorithm. As an example, we present the triangulation of the Barstar protein in Figure 8. By visual inspection, one can see that the representation of the details is still accurate. These results have been obtained using a scale of 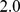 grids per Å. At this scale, the biophysically relevant features (which depend on the smallest atomic radius, usually around 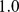 Å), are retained while potentially smaller features are automatically smoothed.

To test the feasibility of our Skin approach for a big system, we performed the construction and triangulation of the 1VSZ adenovirus structure (180,000 atoms), obtaining, at scale = 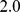 grids per Å, the following figures: i) overall surfacing time: 234 s (17 s for building, 88 s for ray-casting, 129 s for Marching Cubes), ii) peak memory usage: 7.3GB. Incidentally, preliminary runs performed using our development version of NanoShaper, where we are testing the exploitation of more advanced data structures such as the Octrees, show that the memory usage in this case falls to 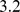
GB.

#### SES: NanoShaper vs EDTSurf and MSMS

**Figure 9:**
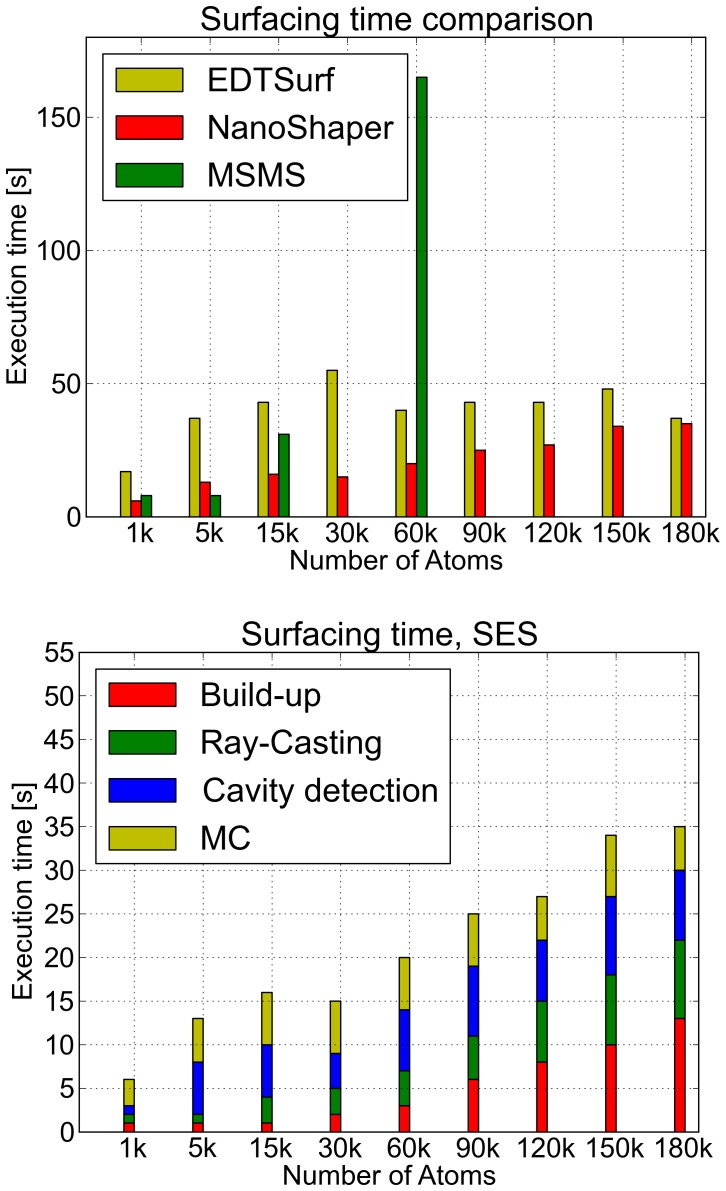
SES: execution times. Performance comparison of the total time needed to build, get cavities, and triangulate the SES for NanoShaper, EDTSurf, and MSMS. Detailed times for NanoShaper are in the lower panel.

As reported in Figure 9, on our benchmark molecules, NanoShaper is faster than EDTSurf which, in turn, is faster than MSMS. For MSMS, there are some missing fields. This is because, if MSMS at some point fails in the process of building a component, either the main one or a cavity, it starts again at a different initial position of the rolling probe. If the failure repeats itself a number of times, MSMS exits. Therefore, although the single calculation is very fast, the whole process can take more time than for EDTSurf and NanoShaper, and it can even not converge. In our tests, EDTSurf never failed and proved to be, in general, a very fast algorithm. It does not scale with the number of atoms but rather with grid size. This explains why the execution time for EDTSurf is almost constant. Indeed, in most of the cases, EDTSurf set up a grid of about 300×300×300.

**Figure 10:**
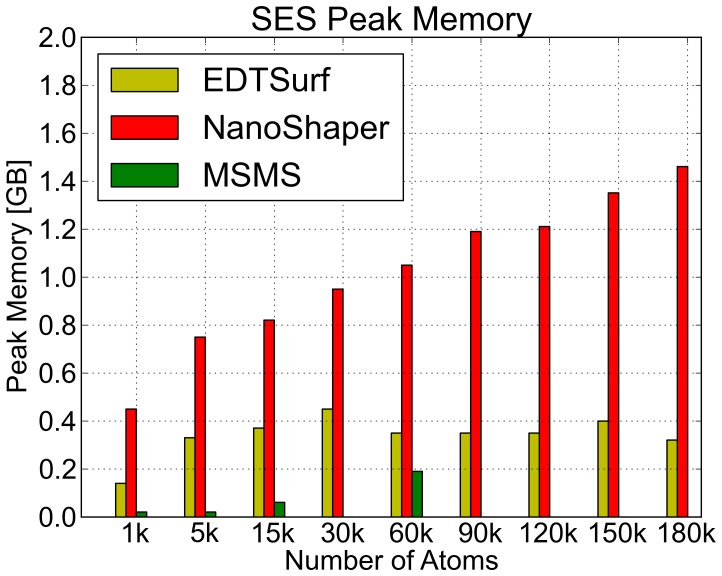
SES: memory requirements. Comparison of the peak memory needed to build and triangulate the SES for NanoShaper, EDTSurf, and MSMS.

In terms of peak memory usage (see Figure 10), MSMS is the less demanding, EDTSurf uses slightly more memory, and NanoShaper, not surprisingly, uses a significantly larger amount of memory. This is due to two factors: patches are kept in memory and analytical intersections are stored using a 3D data structure to allow fast access for the subsequent Marching Cubes step. By employing a sparse 3D structure, such as an Octree, this figure could be significantly reduced. However, the total amount of required memory is still reasonable for a modern workstation.

To assess the numerical accuracy of both the computed area and volume on the main component, we took the calmodulin protein (PDB code 3CLN) as a reference system. We chose this molecule since it has no cavities. We used different scale values, namely 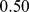, 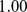, 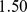, 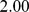, 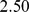, 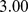, 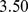, 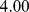, 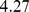 grids per Å. The 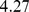 value was chosen because it was the maximum scale allowed by EDTSurf on 3CLN.

**Figure 11:**
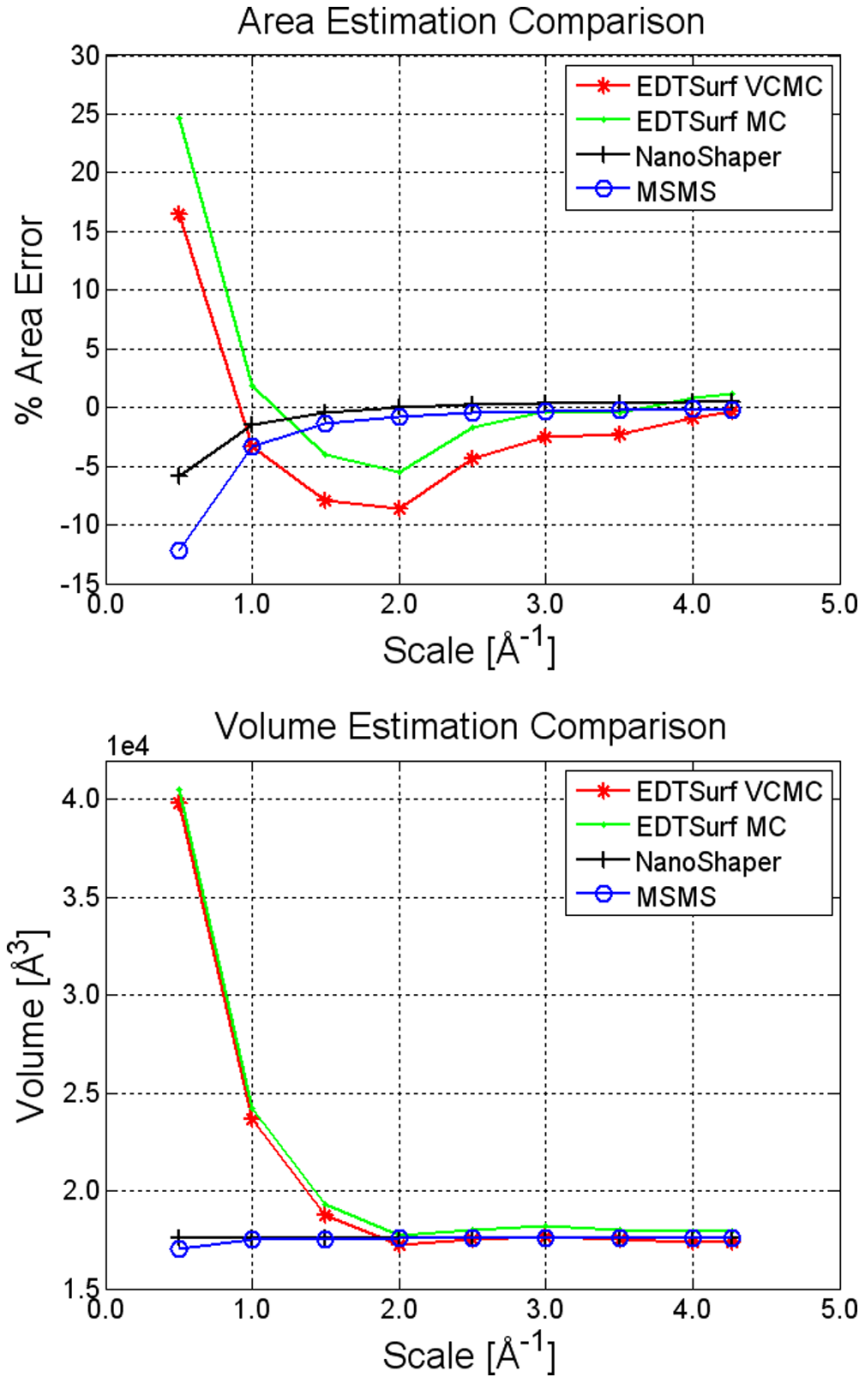
SES: area and volume estimation. Area and volume estimation of the Calmodulin SES made by NanoShaper, EDTSurf, and MSMS at different scale/vertex densities.

Resulting volumes are reported in Figure 11. For the area, we report the percentage relative error with respect to the analytical value computed by MSMS. Results indicate that MSMS and NanoShaper are generally very accurate, both in terms of area and volume, and that NanoShaper is slightly more accurate than MSMS in computing the surface area. Compared to EDTSurf, NanoShaper can get the same accuracy by employing roughly half the scale. One reason could be that the points used for triangulation in EDTSurf do not necessarily lie exactly on the surface. This is due to the Vertex Connected Marching Cubes (VCMC) method and to the fact that, a posteriori, the surface is smoothed without imposing that property. Therefore, our speed-up would be significantly higher if one would keep the accuracy rather than the grid size as a reference figure. These arguments still hold if we use the VCMC rather than the standard Marching Cubes in EDTSurf. As seen in the case of volume calculation, we observe that a scale of 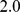 grids per Å provides accurate enough results in the case of area estimation too.

A test was devoted to analyzing the behavior of the different algorithms in the presence of cavities. To this end, we use the FAAH protein. We requested EDTSurf, NanoShaper, and MSMS to compute the number of cavities and their volume. For both EDTSurf and NanoShaper, the scale was changed from 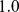 to 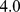 grids per Å by steps of 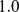 grid per Å. Results in Table 2 show that EDTSurf and NanoShaper provide close results (4 and 6 cavities, respectively) only when the scale is quite high (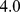 grids per Å). At a lower scale, the number of cavities identified by EDTSurf oscillates. NanoShaper shows a much more stable behavior with respect to resolution. Table 3 gives the detail of the volume of each cavity with varying grid resolutions for NanoShaper. The total volume is stable, as is that of every single cavity.

**Table 2:** Comparison of cavities detection by NanoShaper and EDTSurf when varying grid size.

	NanoShaper		EDTSurf	
Scale[Å 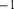 ]	Ncav	Vol [A 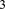 ]	Ncav	Vol [A 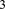 ]
1	6	173.0	3	477.0
2	6	179.2	0	–
3	6	178.3	4	132.0
4	6	178.8	4	162.4

**Table 3:** Detail of the volume of the cavities obtained by NanoShaper on FAAH.

Cav Index	Scale [Å 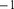 ] 1.0	Scale [Å 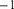 ] 2.0	Scale [Å 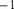 ] 3.0	Scale [Å 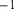 ] 4.0
0	19	21.62	21.48	21.47
1	14	12.37	11.74	11.92
2	41	39.62	39.78	39.73
3	39	42.62	42.00	42.20
4	26	30.25	30.41	30.52
5	34	32.75	32.85	32.91

As a further comparison, we consider the cavities obtained by NanoShaper and EDTSurf at a scale of 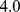 grids per Å together with MSMS: for NanoShaper and EDTSurf, we obtain 6 and 4 cavities with a total volume of 178.8Å^
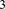
^ and 162.4Å^
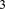
^ respectively, while MSMS gives 11 cavities and a total volume of 893Å^
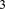
^.

**Figure 12:**
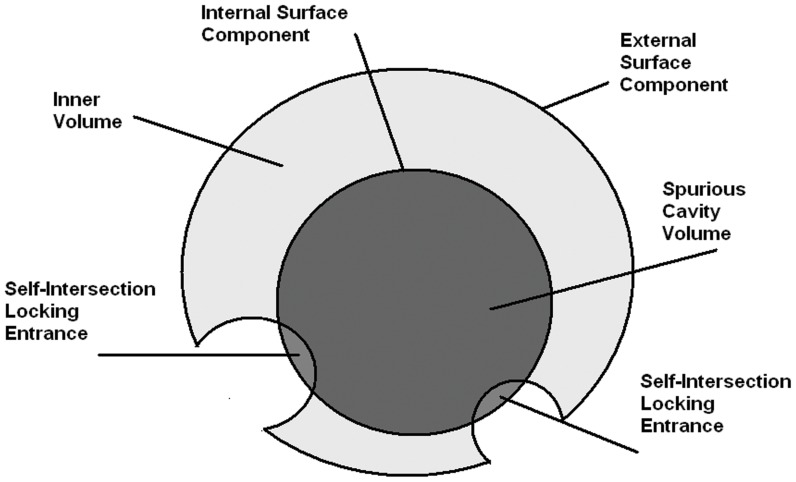
SES: MSMS false positive identification of a cavity. In this figure, a schematic illustration of how the MSMS algorithm can confuse an accessible region with an internal cavity during the SES construction. The probe can roll both inside and outside. In MSMS, this task is performed in two separate steps. If self-intersections occur at the entrance(s) of a given region, they may lead to the depicted situation and to the incorrect detection of a cavity.

While the first two approaches show a certain degree of consistency, the result of MSMS is a bit divergent. In particular, MSMS detects a very big cavity, which is not recognized by the other two programs. Our investigations revealed that this is because, if a self-intersection (see [11]) occurs between the component of a cavity and the main one, then it is not correctly identified, or removed, by MSMS. As a consequence, MSMS misinterprets it and concludes that there is a cavity. This is because, in FAAH, a self-intersection occurs at the entrance of the narrow, but still open, active site. This problem seems to be due to the fact that, in MSMS, every cavity is triangulated independently from both the external component and from those related to the other cavities. The triangulation obtained by aggregating all the triangulated components can, in some pathological cases, be inconsistent. This fact is schematically illustrated in Figure 12. The user must be aware of this possibility because often tunnels are functional regions in a protein and should not be neglected.

As a final test, we computed the SES for the fullerene. Its structure is composed of 60 carbon atoms that lie on the surface of a sphere. This case is challenging from the geometric standpoint because co-sphericity can be a source of numerical instability. The radius of the carbon used was 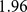 Å, as given by EDTSurf. In contrast to NanoShaper, neither EDTSurf nor MSMS are able to correctly detect the internal cavity. Additionally, we observed that EDTSurf detects a cavity if the probe radius is slightly reduced. This does not occur for MSMS. In EDTSurf, the issue is purely numerical. In MSMS, however, it seems to be structural. What happens in this latter case is that, in fullerene, the atoms that generate the surface of the cavity also generate the main component of the SES. This occurrence seems to be excluded by MSMS, hampering the cavity detection in this case. Interestingly, there can be initial MSMS conditions, namely when the probe starts rolling from within the cavity, that lead to the detection of that cavity. However, such initialization is not under the direct control of the user in MSMS.

#### Surface area estimation

Here we compare our area estimation method to what we term the ‘Incident Ray’ method proposed in [23]. This latter infers the area from the angle 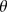 formed by the incident ray and the normal vector at the surface. Let 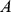 be the area of the plane where 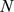 rays are cast, and 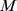 be the number of detected intersections, the estimated surface area is:
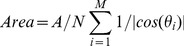
(5)


**Figure 13:**
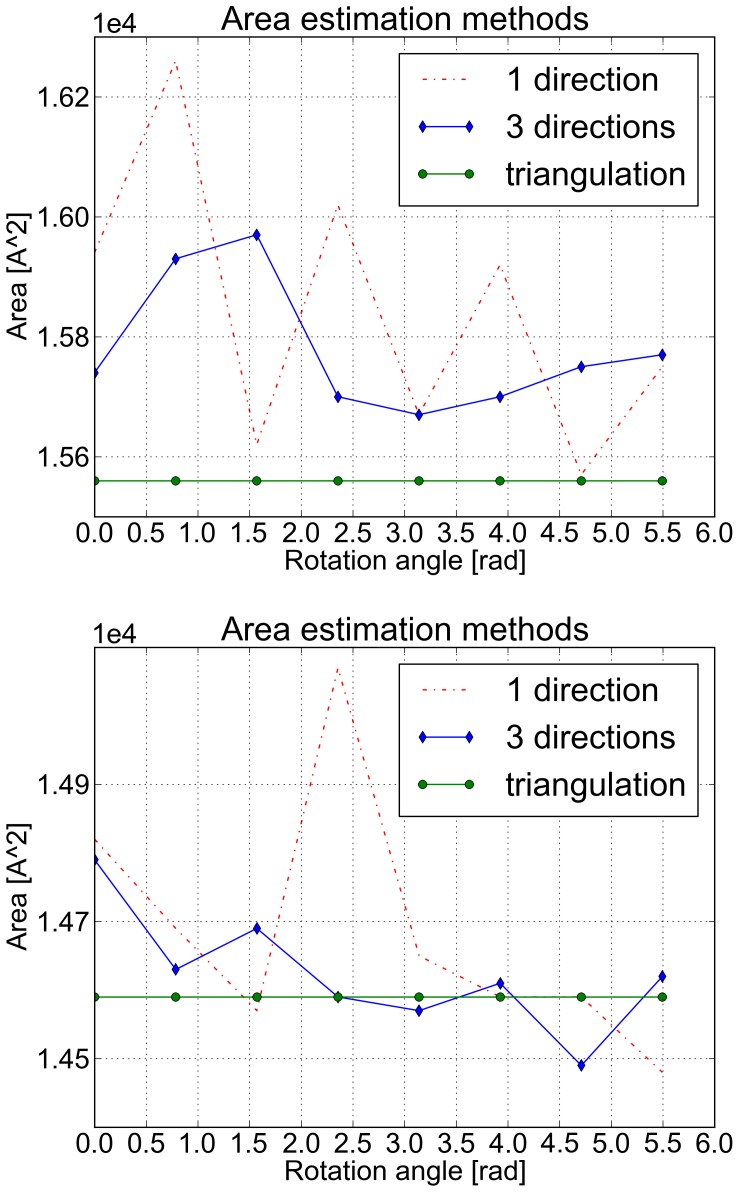
Area estimation: methods comparison. Here, area estimation methods are compared upon rotation of the 1HF0 and 1QA7 molecules.

Due to the division by the cosine, a specific clamping is needed to avoid division by zero errors. By Averaged Incident Ray, we indicate the same method but averaged over three orthogonal incident directions. Finally, by triangulation, we indicate our method. In Figure 13 the results provided by these three methods on the Skin surface area estimation for two proteins (also used in [23]), PDB codes 1HF0 and 1QA7, are reported. We rotated the systems 7 times around their centers by 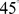 and calculated the area. As can be intuitively expected, the Ray-Casting method is sensitive to molecule orientation. A smaller but still non-negligible dependence is present in the Averaged Incident Ray method, whereas our triangulation algorithm is almost completely insensitive to rotations.

### Application to the Solution of the Poisson-Boltzmann Equation

To validate our method in the context of PDE solution, we interfaced NanoShaper with the well-known DelPhi Poisson-Boltzmann Equation (PBE) solver [21]. PBE rules the electrostatic potential distribution of a system in a solvent with salt at the thermodynamic equilibrium. The DelPhi code solves the PBE on a grid adopting a Finite Difference scheme. It needs the in/out (i.e. solute/solvent) information at the centers of the faces of the grid cubes to assign the dielectric constant representing low and high dielectric regions. To compute all the information needed by the DelPhi engine, the projection of boundary grid points over the surface is also needed [16]. These points correspond to grid cubes that have different faces in different media. To speed up this phase, we built an accelerating auxiliary 3D grid to detect the nearest patches/triangles to a given grid point. The projection allows a more precise calculation of the reaction field energy, a fundamental quantity in this kind of calculation. The importance of the projection resides in correctly placing the polarization charge on the surface (see [16] for more details).

We first evaluated the stability of the reaction field energy with respect to the scale parameter for the Barstar protein, a standard benchmark for this kind of study. As reported in the SI, these tests show that the surface generated by NanoShaper provides accurate results at a resolution lower than the other methods. In another test, we compared both the execution time and the reaction field energy discrepancy between the implemented surface definitions and the internally built DelPhi SES. In this case, triangulation is not required, thus reported times are only about grid coloring and boundary grid point projection. We used, as reference systems, the proteins with the following PDB codes (the given number of atoms is intended after protonation): 1CRN (648 atoms), 1VIS (5080 atoms), 1AFS (19266 atoms), 1GKI (39271 atoms), and 1VSZ (180570 atoms). This latter is a good example of a very large system that can be managed by both DelPhi and NanoShaper. All the molecules were protonated with tleap [36] and the Amber99 force field was used to model radii and charges. As DelPhi settings, we used a perfil of 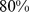 a scale of 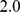 grids per Å (on the 1VSZ structure, the scale was reduced to fit a 513 grid size), zero ionic strength, and dipolar boundary conditions. On the surface side, blobbyness was set to 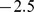, the Skin parameter to 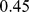, and the SES probe radius was set to 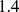 Å. All cavities and voids produced by the original surface definitions were maintained.

**Figure 14:**
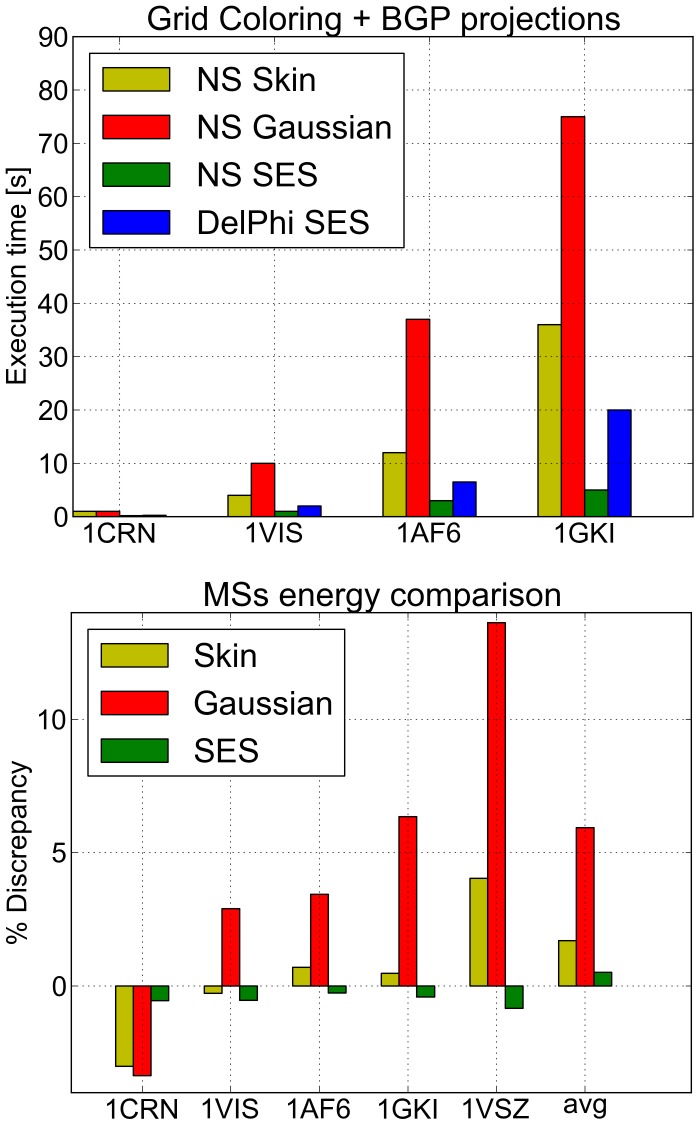
Pre-processing for PDE solution: execution times and accuracy assessment. Comparison of execution times for grid coloring and boundary grid points projection for NanoShaper surfaces and DelPhi. A set of increasingly bigger molecules is used as benchmark. In the lower panel, percentage energy difference of different MS definitions and methods as compared to the DelPhi solver.

From the execution time point of view, results show (see Figure 14) that the SES surface, both in DelPhi and in NanoShaper, is the fastest. We note again that the current Gaussian surface implementation could be further optimized. Despite this, the execution times are still acceptable. For the SES case, we note that our multithreaded version is always faster than the DelPhi internal SES building routine. On the single-threaded version, however, this is true only for molecules with more than 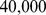 atoms. As expected from previous works [31], [11] the Skin surface is slower than the SES. This is due not only to the quite involved construction phase of the mixed complex, but also to the augmented number of patches that have to be ray-cast. Our tests indicate that the number of Skin patches computed by NanoShaper is about 7 times the corresponding figure for the SES.

For the full 1VSZ structure we obtained the following execution times: Skin 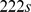, Gaussian 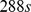, SES 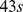, DelPhi 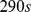.

From the point of view of the physical model agreement (see again Figure 14), the Gaussian surface shows the most significant discrepancy with respect to the DelPhi SES energy. The Skin surface tracks the energy of DelPhi SES quite well, with a maximum error of 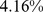. The SES surface computed by NanoShaper gives results extremely similar to those obtained by the DelPhi SES with a maximum error of 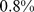 and an average error of 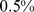.

## Discussion and Conclusions

In this work, we presented a general framework specifically aimed at triangulating and processing surfaces of nano-sized systems in solution. It is intended to be extremely flexible, in the sense that virtually any surface definition can be supported, provided that a surface/ray intersection algorithm can be devised for it. The main functionalities considered in this framework are grid-consistent triangulation, volume and area estimation, cavity identification, and grid coloring for subsequent numerical solution of partial differential equations. We also made an implementation of this framework (called NanoShaper), which is able to build the molecular surface according to the most widely used definitions. We first validated our method by assessing its stability and robustness with respect to resolution. Then, we compared our approach to the most widely used and better performing alternatives. Specifically, these were: the Krone and Lindow techniques for building the SES [25], [31], the Lindow approach for building the Skin surface [31], the CGAL Kruithof algorithm [9] for triangulating the Skin surface, and, finally, the EDTSurf [18]and MSMS [14] methods for triangulating the SES. Moreover, we integrated NanoShaper into the DelPhi Poisson-Boltzmann solver and compared the electrostatic energies of some molecules with those obtained by the original method [16].

Results show that the proposed framework is a viable solution for triangulating an arbitrary closed surface and coloring a grid for a successive PDE solution in a fast and accurate way.

From the robustness and accuracy standpoints, very good figures were always achieved. The ray-casting method used in NanoShaper is not iterative, therefore it reaches a solution deterministically. Additionally, it computes the surfaces in full respect of the underlying theoretical definitions and adopting analytical calculations whenever possible.

As per generality, let us consider the method used in DelPhi [16] for the SES. This method is inherently based on the computation of distances and projections. It is thus highly efficient when dealing with spheres, but its performance could deteriorate when dealing with more complex surface patches. This observation is supported by the experience that the Authors had when extending it to the Skin surface, for which a projection primitive can be given [11]. In contrast, the present method is based on intersections rather than projections, which results in higher generality and larger numerical stability. Most of these concerns apply also to EDTSurf [18], which relies on a distance-based algorithm and on the fact that the fundamental primitive of the SES is the sphere. The same argument holds for MSMS [14] and the Akkiraju and Edelsbrunner SES triangulation algorithm [37]. Based on these observations, we expect that a numerically robust generalization of these algorithms to other surface definitions involving more complex patches would be far from trivial.

Table 4 summarizes the features of the triangulation/grid coloring algorithms considered in this work. The proposed triangulation algorithm generally succeeds in achieving a good performance. It also exploits the multithreading feature of current multicore CPUs in the ray-casting phase. In particular, our Skin triangulation algorithm is more than one order of magnitude faster than that of Kruithof [9] and has much lower memory requirements. This is achieved by setting a grid resolution based on the smallest atomic size and avoiding the triangulation of surface details that would be irrelevant from the standpoint of the physical model. Another alternative, the Cheng and Shi Skin surface can be triangulated only for 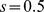 [7] whereas in Kruithof’s and in our approach the user is free to choose the desired shrinking constant.

**Table 4:** Comparison of some molecular surface building algorithms; interp. means that triangulation vertices are interpolated via the Marching Cubes algorithm.

Algorithm	Cavities	Precision	Surface Definitions	Parallel	Iterative
EDT [18]	yes with removal	interp	SES, SAS, VdW	no	no
MSMS [14]	yes, some spurious	analytical	SES, SAS, VdW	no	yes
Akkiraju [37]	yes	analytical	SES, SAS, VdW	yes	no
DelPhi [16]	yes	semi-analytical	SES, SAS, VdW	no	yes
LSMS [15]	yes	interp	SES, SAS, VdW	no	yes
NanoShaper	yes with removal	analytical	any manifold surface	yes	no
Kruithof [9]	no	analytical	Skin	no	no
Gamer [33]	no	interp	Gaussian	no	no

Our SES presents a timing performance similar to that of EDT [18] and MSMS [14] for triangulation and similar to [31] and [25] for build-up over a common set of molecules ranging from 2,000 to 100,000 atoms. However, our Skin build-up is faster than the single-threaded version shown in [31].

Our approach is inherently grid-based. One way to increase speed and reduce memory requirement is thus by reducing the scale. Using a scale of 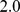 grid per Å is usually reasonable from the point of view of the physical model. At that scale, we were able to build and triangulate the SES and Skin surfaces of a protein of 180,000 atoms on a standard workstation.

In NanoShaper, a non-optimized version of the Gaussian surface was implemented: this surface has been proposed for biophysical computations in [38]. A recent work of Juba and Varshney [22] showed an effective algorithm to estimate its surface area. In [22], the rays, called *lines*, are cast within a sphere that encloses the molecule. The method in [22], similarly to ours, can be used to measure the area of any manifold surface. In that particular implementation, the Gaussian surface was used and the obtained results confirm the discrepancy between that surface and the SES in terms of surface area (up to 17.92%). From the algorithmic standpoint, ray casting is not used to triangulate in either [23] or [22].

The ray-casting procedure is inherently parallel since each ray is independent of the others. Therefore it could benefit from a GPU-based implementation. In this regard, the timing results in [20], [22], [25], [31], [39], [40] are very encouraging. Ray casting (where each ray stops at the first visible intersection) and marching cubes routines have already been developed on this architecture [25], [30], [31], and it would be intriguing to see the results of our 2D acceleration data structure on a GPU device. A possible pitfall in naively moving our procedure to GPU could be the lack of regularity, since different rays can have a very different number of intersections, leading to an unbalanced workload. Alternatively, the presented approach is very suitable for exploiting the features of the Xeon Phi architecture. It would be very interesting to assess the parallel scalability on this platform.

Our implementation is available at the web site www.electrostaticszone.eu, together with several graphical Python scripts and a Python binding which provides access to all the NanoShaper classes.

Possible developments of this work are: the possibility of mixing molecular systems with geometric objects in the perspective of multiscale coarse-grained modeling, the implementation of a parallel Marching Cubes algorithm, the possibility of acquiring surfaces from variegate experimental data, the possibility of tracing cavity evolution in Molecular Dynamics trajectories, and the reduction of the memory footprint by exploiting suitable data structures such as Octrees, which is already under development and is providing promising preliminary results.

## Supporting Information

File S1 **Mathematical details concerning the SES and the Skin surface definition and construction.** Some algorithmic improvements aiming at increasing the performance of NanoShaper: cure for the occurrence of inconsistent configurations after cavity removal; parallelization and acceleration grid. Stability tests for reaction field calculation in the PBE solution. Interactive figures illustrating the differences between the SES built by MSMS and that built by NanoShaper.
(PDF)
