# Supplementary material for: A general and Robust Ray-Casting-Based Algorithm for Triangulating Surfaces at the Nanoscale
Source: PLoS One. 2013 Apr 5;8(4):e59744. doi: 10.1371/journal.pone.0059744 (PMC3618509; doi:10.1371/journal.pone.0059744)
Supplement: File S1 — Mathematical details concerning the SES and the Skin surface definition and construction. Some algorithmic improvements aiming at increasing the performance of NanoShaper: cure for the occurrence of inconsistent configurations after cavity removal; parallelization and acceleration grid. Stability tests for reaction field calculation in the PBE solution. Interactive figures illustrating the differences between the SES built by MSMS and that built by NanoShaper. (PDF) [file pone.0059744.s001.pdf]

Supporting Information
"A general and robust ray-casting-based algorithm for triangulating surfaces at the nanoscale"

Algorithmic details on SES construction

According to Alpha Shapes theory, a specific subset of the Delaunay triangulation (named the alpha shape complex) of the set of atoms that constitute the molecule has to be computed, after increasing their radius by that of the probe. The obtained complex is a set of points, edges and facets. It is a sort of *skeleton* of the SES, so that each point corresponds to an exposed atom, each edge corresponds to a toroidal patch (i.e. a blending patch between two atoms), and each facet (triangle) corresponds to a re-entrant patch of the SES (i.e. a spherical triangle generated by the probe while it rolls over the concave portions of the solute); each simplex can be classified in interior, exterior or singular. An example of the patches/simplices produced by the Alpha Shape is shown in figure S1.

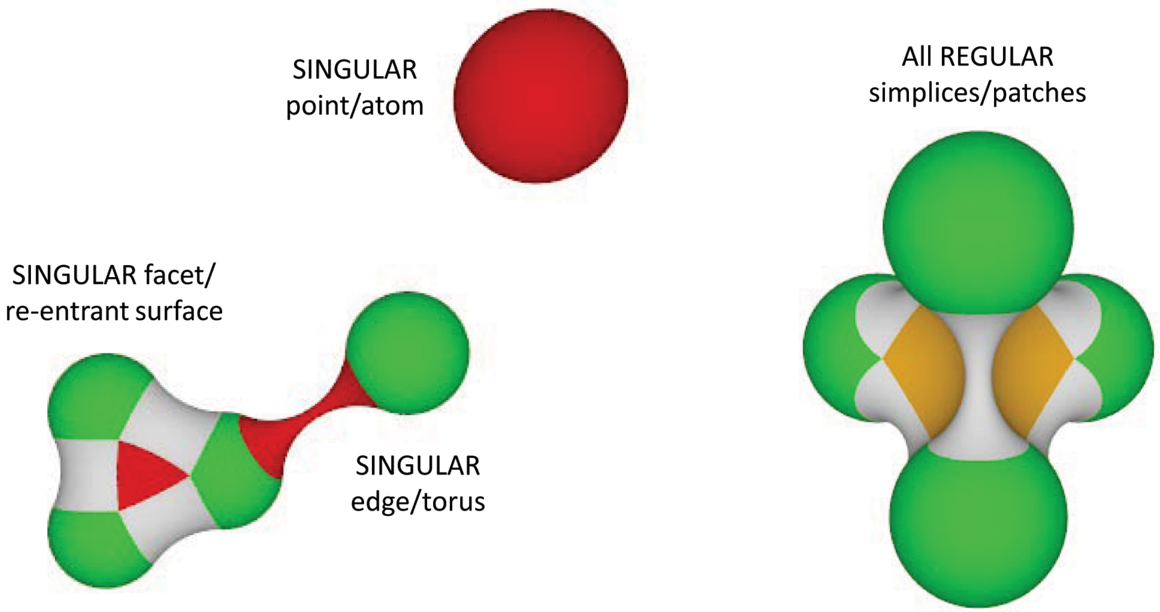

Figure S1. Singular and Regular simplices. In red Singular simplices/patches of the alpha shape/SES, in other colors the Regular ones.

Unfortunately, the alpha shapes theory provides no information about self-intersections (see [1]). These can occur between different re-entrant patches or when the major radius of a torus is less than the radius of its circular section. In our framework, the treatment of self-intersections is worked out in a successive processing step.

Algorithmically, the SES computation can be decomposed in the following steps:

1. Load atomic positions and radii and increase these latter by the probe radius value
2. Compute the Delaunay Regular Triangulation (3D)
3. Apply the alpha shape Triangulation filtering using $\alpha = 0$

4. For each filtering-survived vertex, edge, facet, get the corresponding equation and trimming solids (spheres, planes)

5. Remove the self-intersections

6. Build the auxiliary grid and finally ray-trace

The remaining steps are as in the usual workflow (e.g. cavity detection, triangulation).
To explain how the equations are worked out, we need to classify the simplices such as vertices, edges, and facets (triangles):

- A simplex is called INTERIOR if it does not contribute to the SES.
- A simplex is called REGULAR if it contributes to the SES and it is not isolated.
- A simplex is called SINGULAR if it contributes to the SES and it is isolated.

By isolated/SINGULAR vertex, we intend a vertex nonconnected to any other vertex via an edge. By isolated/SINGULAR edge, we intend the segment connecting two atoms for which the probe can freely roll without ever getting locked during its rotation by any other third atom. The simplest instance of this case is a two-atom molecule. By isolated/SINGULAR facet, we intend a facet that produces simultaneously two symmetric reentrant patches.
In particular, all exposed atoms are either REGULAR or SINGULAR, all the isolated/non-isolated toroidal patches are SINGULAR, REGULAR edges, respectively, and all isolated/non-isolated re-entrant patches are SINGULAR/REGULAR facets, respectively. Figure S1 graphically explains the SINGULAR and REGULAR distinction on the SES.
The patch/trimming equations computation and candidate self-intersecting patches selection tasks can be decomposed in the following substeps:

1. For each alpha shape vertex if it is not INTERIOR, then compute a first set of trimming spheres by using information on its neighboring buried atoms (two atoms are neighbors if they share an INTERIOR edge). A second set of trimming spheres will be computed together with the toroidal patches.

2. For each SINGULAR/REGULAR facet, the probe position is computed together with the three trimming planes that delimit the spherical triangle patch. If that patch is REGULAR and if the spherical triangle intersects the Delaunay facet, the former is added to the list of patches to be checked for self-intersections. One very useful aspect that is used in the Poisson-Boltzmann solver is that the calculated patches keep memory of the atoms that produced them.

3. For each REGULAR/SINGULAR edge, compute the equation of a torus, as well as its trimming spheres and trimming planes. A torus can be self-intersecting. It is called self-intersecting if the major radius of the torus is smaller than the minor radius, leading to a spindle torus. In this case, a trimming sphere is added to cancel out the portion of space that delimits the spindle torus. Trimming spheres delimit the torus along the Delaunay edge, while trimming planes cut the torus in its visible sections. When the torus comes from a SINGULAR (isolated) edge, the trimming planes computation can be skipped. After this phase, each torus is linked to the atom pair from which it was generated. The trimming sphere of each torus is also used to trim exposed atoms. After this phase, the information related to both tori and exposed atom patches is complete.
   Finally, if a torus is self-intersecting, the two adjacent re-entrant spherical patches are added to the self-intersection list. The fact that a torus is self-intersecting is a necessary condition for the self-intersection of re-entrant patches [2].

On the Skin surface construction

The basic entity underlying the *Skin* surface is the *mixed complex.* It is composed of *mixed cells* μ_X^s. These are solids that can be computed starting from the additively weighted Voronoi diagram or its geometrically dual Delaunay tetrahedrization [3]. The mixed cell is defined as the Minkowsky sum (indicated by "$\oplus$" and ruled by s) of a Voronoi cell with its corresponding Delaunay cell:

$$\mu_X^s = \{s \cdot \nu_X \oplus (1-s) \cdot \delta_X\} \tag{1}$$

The mixed complex partitions the 3D space in convex nonoverlapping polyhedra and, for $s = 0$ and $s = 1$, it coincides with the Delaunay Tetrahedrization and the Voronoi diagram, respectively. All patches are quadric surfaces, either hyperboloids or spheres. Each patch is trimmed by the corresponding mixed cell. Figure S2 represents an example of *Skin* surface with color-coded patches. In the *Skin* building process,

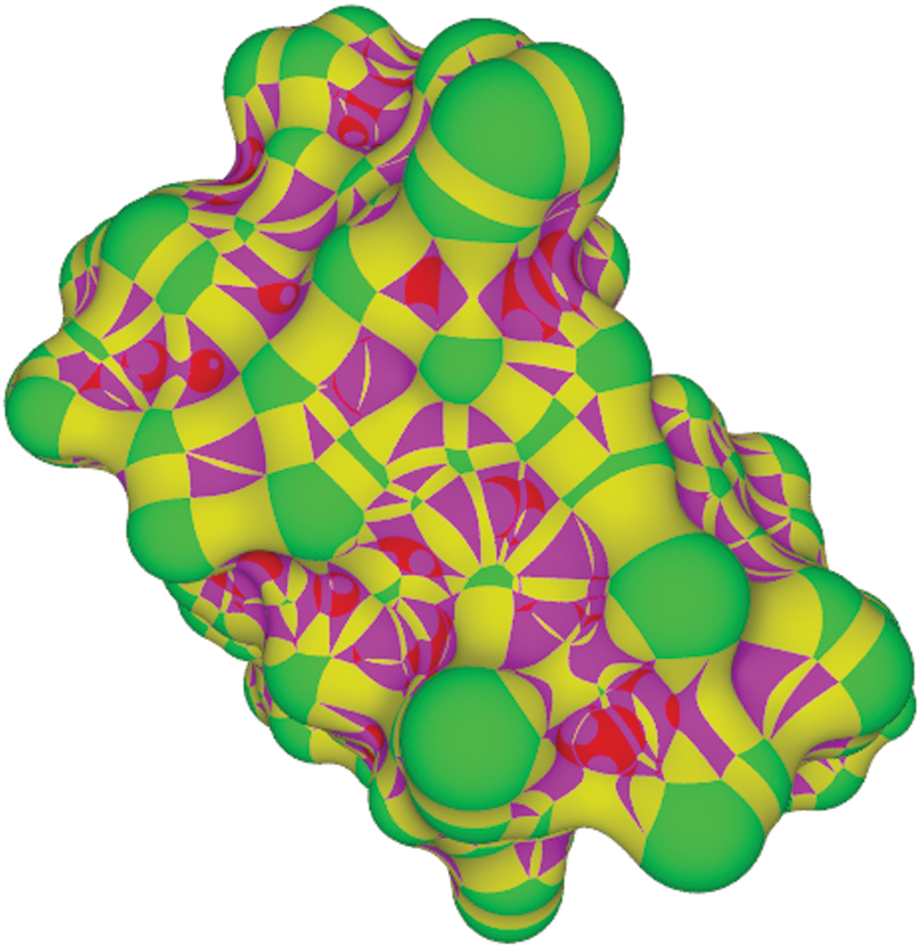

Figure S2. Skin surface. The skin surface of a protein with highlighted patches ray-traced by PovRay: yellow and magenta patches are hyperboloids (respectively generated by Delaunay Edge + Voronoi Facet and Delaunay Facet + Voronoi Edge), in green the atoms (reduced Voronoi polyhedra) and in red concave spheres (reduced Delaunay tetrahedra).

the first step is to compute the mixed complex from which the patch equations can be derived. To this

end, we built the regular Delaunay tetrahedrization and its dual, the weighted Voronoi diagram. Both procedures need careful management of floating point errors. For this, we used the CGAL C++ library [4]. To manage ideally infinite Voronoi regions, we used a parallelepiped as bounding box and added suitable dummy atoms before processing. After the Voronoi diagram is computed, the mixed complex and the surface equations are computed analytically [1] by navigating on the regular triangulation. From this information, a ray-tracing based triangulation can be computed. Figure S2 shows an example of Skin surface in PovRay format. To perform the clipping of each quadric patch during the ray tracing, we used the original mixed complex solids. Moreover, the regularity (axis alignment) of the traced rays allows the definition of a cache buffer where the partial results of ray-to-quadric intersections can be stored to speed-up the computation. Before the ray-tracing procedure was performed, the 2D accelerating grid was computed as previously described.

Inconsistent configurations after cavity removal

Cavity removal can lead to inconsistent configurations. This can occur when a small invagination on the surface is erroneously detected as a cavity due to low grid resolution. If NanoShaper fills this presumed cavity, it can happen that two vertices on the same edge of the grid have a different in/out status, but no intersection between them has been detected (see figure S3). Our implementation is able to detect this situation and to avoid the creation of a nonmanifold, i.e. pierced, surface. We observed this phenomenon only at low resolutions when employing intricate surfaces such as the Skin. The overall impact of our correction approach on the surface was however minimal due to its local character.

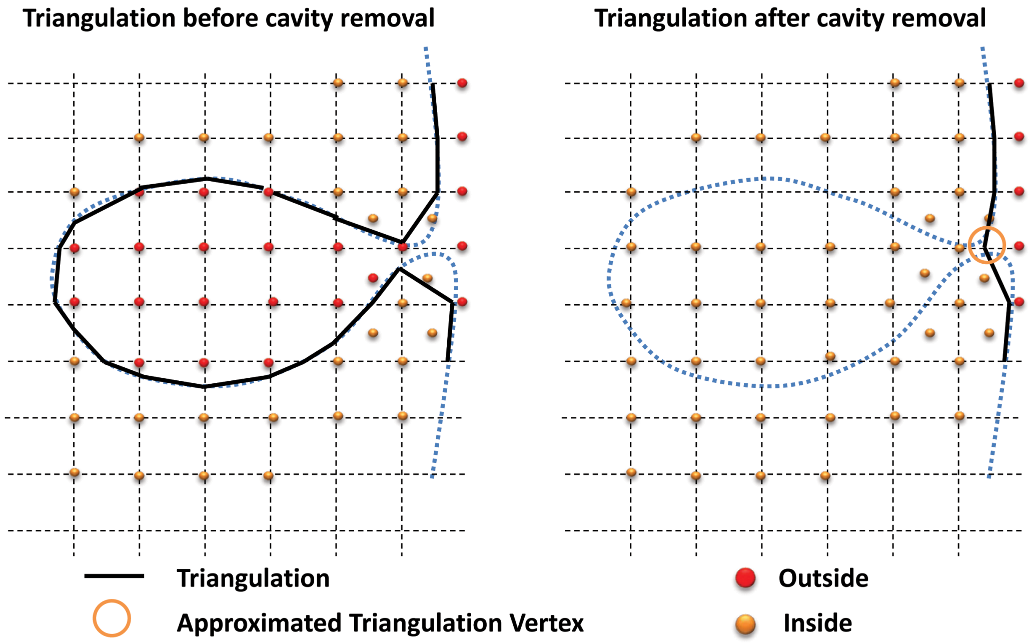

Figure S3. 2D example of a criticality in cavity detection and removal. Due to low grid resolution, a superficial invagination can be marked as an internal cavity. If the algorithm is set to fill that cavity, an inconsistent situation can occur, since one or more vertices that would close the cavity are actually lacking. In order to avoid such inconsistency, NanoShaper introduces an additional vertex at the midpoint along a cube edge and closes the surface. The final surface is watertight.

Implementation details

Parallelization

All ray castings are independent, thus our technique is inherently and trivially parallelizable. In our implementation, which is a portable C++ code, we parallelized the ray-casting procedure using the Boost threads. We chose Boost threads because they are platform-independent middle tier and assure code/performance portability and the handling of complex parallel tasks. Boost threads have the further advantage that they do not require a particular compiler support because they belong to the Boost library. This parallelization assures an ever-increasing scaling so long as an increasing number of cores are embedded in the same CPU die. Interestingly, this method can be ported to GPUs.

Acceleration grid

The rationale behind the 2D data structure instead of a usual 3D grid is the following: in *generic* ray-casting the direction of rays is not known a priori, in the current case ray directions are always parallel to coordinate planes; this fact can be used to increase performance. This is possible since while in a 3D grid, the same patch can be mapped to different grid cubes that are consecutive along the same direction of a ray, this cannot occur if a 2D data-structure is used (which basically projects all the patches along a direction on a square). In this way, multiple intersection tests are avoided and performance increases. Looking at the data structure in figure S4 one can see that for the first vertical ray the only patches to be tested for intersection are A2 and A1. Using a 2D data structure, these patches are tested only once whereas using a 3D data structure each patch is mapped to 3 grid cubes, and requires 3 tests for intersection.
Another, more technical, reason for improved performance is that this technique is a sort of complete loop unrolling. Indeed in the 3D case one has a loop over the grid cubes on the ray direction nested with a loop over the patches. In the 2D case one has only one big loop over all the patches. Eliminating one nesting level makes the code more prone to be optimized by the compiler. Figure S5 compares the ray casting times for the 2D and the 3D data structures with varying triangles number.

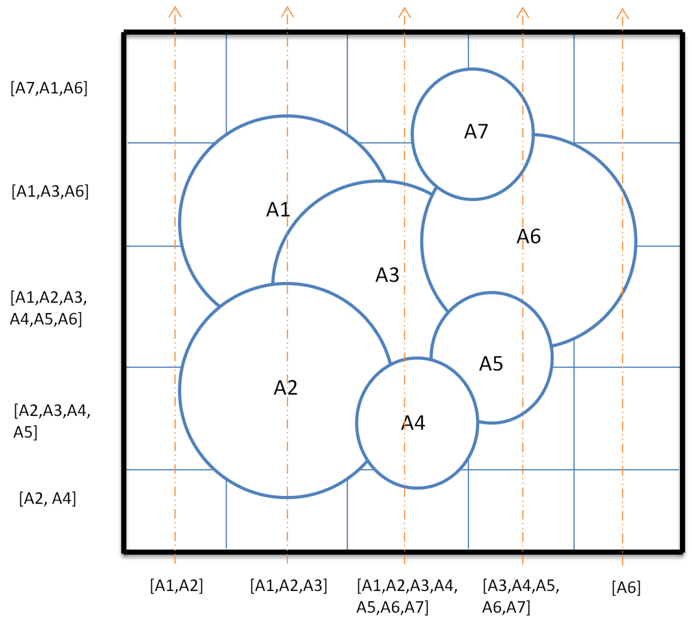

Figure S4. Acceleration data structure. Before ray-casting from each coordinate plane (actually a face of the grid, here shown as a cross-section), the set of patches along the ray direction are collected to accelerate successive ray traversal.

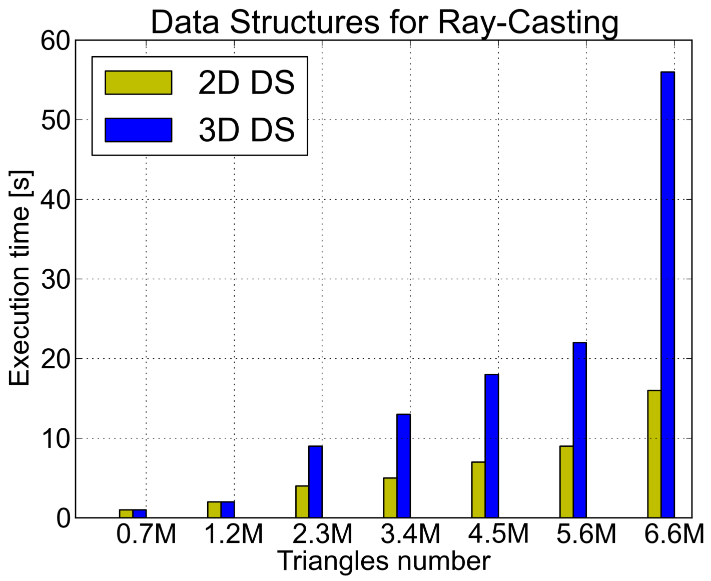

Figure S5. Data structure comparison. Comparison between 2D and 3D acceleration grids used for triangulated mesh ray-casting.

Random ray perturbation

As explained in the main text, in order to obtain a robust ray/surface intersection procedure, we perform a checksum on the number of intersections. In case the number of intersections is odd, we re-cast the ray using an equal displacement of both ends, so as that the direction of the ray remains unchanged. The amount of the perturbation, i.e. 10^{-3} Å , was chosen because it is two orders of magnitude smaller than the accuracy which is typically used for atom radii. Therefore, a grid spacing of 0.1 Å would corresponds to a scale of 10.0 $Å^{-1}$, which is 5 times the scale usually adopted in this application. Based on this hypothesis, we can safely assume that the re-cast ray will not exit the grid cube from which it was originally cast.

Stability tests for reaction field calculation

We evaluated the stability of the reaction field energy with respect to the scale parameter for the Barstar protein, a standard benchmark for this kind of studies. For this calculation, we used all the available surface definitions in NanoShaper. As shown in figure S6, the DelPhi internal SES algorithm is in excellent agreement with NanoShaper SES and Skin. It is in good agreement also with the meshed SES created by MSMS. Consistently with what we observed in [1], it is less in agreement with the results coming from the use of the Gaussian surface.

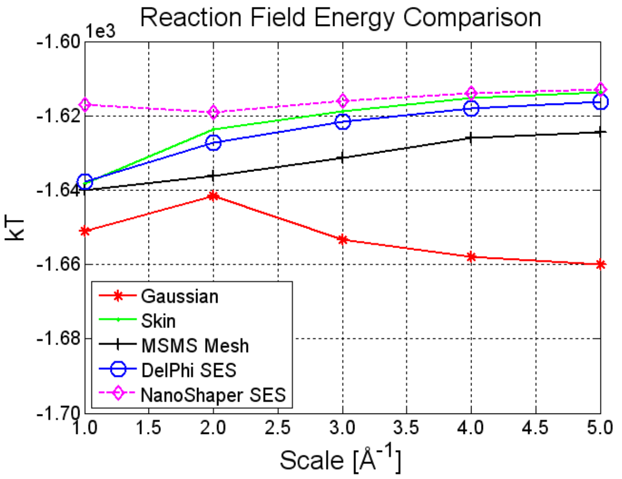

Figure S6. Stability in PDE solution: electrostatic reaction-field energy estimation. Analysis of the reaction-field energy of the Barstar protein immersed in solution. This quantity is calculated by solving the Poisson-Boltzmann equation over a grid that was previously colored by using NanoShaper, at different scales and using different surface definitions.

MSMS Vs NanoShaper surface comparison

The last figure (figure S7) of this SI contains two interactive 3D images. Two SES representations of the molecular surface of the FAAH protein are shown. The first is produced by MSMS, one of the most widespread molecular surface visualization tools, and the other by our implementation, NanoShaper. Some artifacts that are present in the MSMS representation, and that originate from an erroneous treatment of self-intersections, can be duly appreciated.

Figure S7. Interactive MSMS (upper panel) and NanoShaper (lower panel) Connolly Surfaces. This interactive 3D image format can be visualized by the standard free Acrobat pdf Reader (to produce similar interactive figures, one can use the off2pdf.py script at the site http://www.electrostaticszone.eu/index.php/downloads/cat_view/2-scripts-and-utilities). The details that differ between the two representations can easily be visualized by selecting View 1 in the Views menu of the 3D object. This menu appears if one moves the mouse over the 3D object. (It is possible that Acrobat reader asks for permission to start the representation and it is also possible that one has to click on the figures to start the view).

References

1. Decherchi S, Colmenares J, Catalano C, Spagnuolo M, Alexov E, et al. (2013) Between algorithm and model: Different molecular surface definitions for the Poisson-Boltzmann based electrostatic characterization of biomolecules in solution. Communications in Computational Physics 13: 61-89.

2. Krone M, Bidmon K, Ertl T (2009) Interactive visualization of molecular surface dynamics. IEEE Transactions on Visualization and Computer Graphics 15: 1391–1398.

3. Edelsbrunner H (1999) Deformable smooth surface design. Discrete and Computational Geometry 21: 87-115.

4. Boissonnat JD, Devillers O, Pion S, Teillaud M, Yvinec M (2002) Triangulations in cgal. Comput Geom Theory Appl 22: 5-19.
